# Respiratory pathogens in infants and young children with acute respiratory illness: a prospective cohort study, China, 2023–2024

**DOI:** 10.3389/fpubh.2025.1548190

**Published:** 2025-08-04

**Authors:** Yuqing Li, Mingwei Wei, Lunbiao Cui, Xiang Huo, Lisha Ma, Ran Tao, Tao Wu, Suyang Qi, Baolong Wang, Xiuyun Shi, Yuanbao Liu, Wenqing Liu, Yiyue Ge, Li Chen, Xiujuan Zhao, Jingjing Wu, Runjie Qi, Fengcai Zhu, Jingxin Li

**Affiliations:** ^1^School of Public Health, National Vaccine Innovation Platform, Nanjing Medical University, Nanjing, China; ^2^Jiangsu Provincial Medical Innovation Center, National Health Commission Key Laboratory of Enteric Pathogenic Microbiology, Jiangsu Provincial Center for Disease Control and Prevention (Jiangsu Provincial Academy of Preventive Medicine), Nanjing, China; ^3^School of Public Health, Southeast University, Nanjing, China; ^4^Sucheng District Center for Disease Control and Prevention, Suqian, China; ^5^Suyu District Center for Disease Control and Prevention, Suqian, China; ^6^Siyang County Center for Disease Control and Prevention, Suqian, China

**Keywords:** acute respiratory infections, infants and young children, cohort study, targeted next-generation sequencing, pathogens

## Abstract

**Background:**

Infants are vulnerable to acute respiratory infections (ARIs), which result in pediatric morbidity and even fatalities. A surge of ARIs among infants and young children was reported in China in 2023, garnering global attention. We aimed to investigate the frequency and types of respiratory pathogens associated with the risk of acute respiratory illnesses in infants and young children during this period.

**Methods:**

We established a cohort of 2-month-old healthy infants across three counties in Suqian City, Jiangsu Province, China. The infants in the cohort donated throat swabs at enrollment and then underwent a follow-up for active monitoring of common ARIs. Throat swabs collected from infants experiencing common ARIs were analyzed for respiratory pathogens using targeted next-generation sequencing (tNGS).

**Results:**

Between 7 February and 17 April 2023, a total of 804 infants were invited to participate. Among them, 796 participants were enrolled (423 [53.1%] male, 373 [46.9%] female) with a median age of 71.0 days. Frequently detected respiratory pathogen carriers at baseline included *Staphylococcus aureus* (*S. aureus*) (17.9%), Cytomegalovirus (CMV) (16.0%), and *Acinetobacter baumannii (A. baumannii)* (15.3%). In the follow-up period up to 30 April 2024, 1,412 episodes of common ARIs were recorded (164.5 per 100 person-years). A total of 675 specimens were collected during the episodes of common ARIs and qualified for analysis, of which 636 (94.2%) had at least one positive detection of respiratory pathogens, with the most frequently detected pathogens being CMV (43.7%), *A. baumannii* (35.6%), and Rhinovirus (HRV) (29.3%). Among the pathogens, human adenovirus (HAdV) and human respiratory syncytial virus (RSV) (18, 36.0%) were the most common pathogens detected among 50 hospitalized cases of ARIs.

**Conclusion:**

Our data provide a better understanding of epidemiological patterns of respiratory pathogens in infants and young children from 2023 to 2024. CMV, *A. baumannii,* and HRV were the most commonly detected pathogens in common ARI cases, while HAdV and RSV were more frequently observed in hospitalized cases with ARIs. These findings suggest that shifts in the pathogen spectrum are closely linked to disease severity, highlighting the need for targeted prevention and control strategies.

## 1 Introduction

Acute respiratory infections (ARIs) represent a significant contributor to global morbidity and mortality associated with infectious diseases worldwide, particularly in infants and young children who are more susceptible to severe cases of ARIs (1, 2). According to the World Health Organization (WHO), ARIs remained a leading infectious cause of deaths in children under 5 years of age, accounting for approximately 740,000 fatalities in 2019, representing 13.9% of all deaths in this age group (3). ARIs can be caused by common respiratory pathogens such as human respiratory syncytial virus (RSV), rhinovirus (HRV), influenza A/B viruses, human adenovirus (HAdV), *Bordetella pertussis* (*B. pertussis*), and *Haemophilus influenzae* (*H. influenzae*), as well as less common pathogens such as enterovirus (EV) and coxsackievirus A (CVA) (4, 5). Continued ARI surveillance is critical to determine the prevalence of respiratory pathogens, elucidate risk factors, health disparities, and guide prevention policies.

In 2023, China reported a surge of ARIs in infants and young children, which is accompanied by a significant increase in outpatient consultations and hospital admissions, garnering global attention (6). In fact, many countries experienced a rebound of common respiratory diseases in the first winter after the relaxation of COVID-19 prevention measures (such as mask-wearing and travel restrictions). For instance, in November 2022, the United States saw the highest number of hospitalizations due to influenza since 2010 (7). After investigation, the WHO confirmed that China had not detected any abnormal or novel pathogens, attributing the increase in respiratory diseases to multiple known pathogens (8). Experts have suggested that the 3 years of stringent COVID-19 measures heightened people’s susceptibility to respiratory infections, which may have contributed to the higher incidence of respiratory diseases this year. This phenomenon is referred to as the immunity gap, indicating that prolonged low pathogen exposure leads to a greater proportion of susceptible individuals, thereby increasing the likelihood of future outbreaks (9). However, the disease wave in China differs somewhat from that in other countries. While many regions struggled with influenza and RSV infections during the post-pandemic winter, China witnessed a surge in *Mycoplasma pneumoniae* (*M. pneumoniae*) cases, with the resistance rate to macrolide antibiotics increasing from 70 to 90% (10). It is highly worthwhile to analyze the infection status and epidemiological characteristics of common pathogens causing ARIs in infants and young children in China during 2023–2024. However, limited information was available on the epidemiological patterns of respiratory pathogens among infants in China during this period.

Cohort-based surveillance combined with etiological identification is essential for the early detection of increases in ARIs and is important to determine the pathogen, guide clinical treatment, and prevent and control epidemics (11, 12). Recently, multiplex molecular biology techniques have facilitated the swift identification of respiratory viruses across various settings, replacing the conventional approaches for diagnosing respiratory tract infections, such as cultures, direct fluorescent-antibody staining, and enzyme immunoassays (11, 13). Targeted next-generation sequencing (tNGS) is a new generation sequencing technology that combines multiplex polymerase chain reaction (mPCR) with high-throughput sequencing (14). The applications of tNGS provide evidence for pathogen identification, detect potential resistance or virulence genes, and are used for molecular typing, pathogen tracing, and epidemiological research (15, 16). Additionally, this study utilizes multiplex PCR technology for the serotyping of *Streptococcus pneumoniae (S. pneumoniae)*. Compared to traditional serological methods, multiplex PCR offers higher throughput and is more cost-effective than whole-genome sequencing, making it particularly suitable for large-scale sample screening. However, this technology has certain limitations, including primer interference and restricted serotype coverage, such as its inability to detect new variants (17). Nevertheless, given its high applicability and alignment with the research objectives, multiplex PCR is well-suited to explore the distribution of major *S. pneumoniae* serotypes in the region.

Here, we reported a prospective cohort study for the surveillance of common ARIs among infants in China, from 2023 to 2024, to reveal the prevalence and epidemiological characteristics of respiratory pathogens and to explore the associated risk factors for the positive detection of certain pathogens. This was, to our knowledge, the first cohort study focusing on infants and children to explore the epidemiological characteristics of respiratory pathogens during this period in China. Our study provides important insights into the epidemiological characteristics of ARIs and the associated respiratory pathogens in infants and children during 2023–2024 in China, offering valuable knowledge for informing public health policies and enhancing the pandemic response.

## 2 Methods

### 2.1 Study design and participants

We recruited infants aged 2 months as participants at three vaccination clinics in Suqian City, Jiangsu Province (Suqian District, Suyu District, and Siyang County) when the parents took infants to the clinics for routine vaccination according to China’s expanded program on immunization or health examination. The recruiting was conducted from 7 February to 17 April 2023. Healthy infants, boys or girls, aged between 60 and 90 days at enrollment, were invited to participate in a surveillance of common ARIs. Infants with acute illness, severe chronic disease, acute onset of chronic disease, congenital malformations, genetic defects, severe malnutrition, with a history of epilepsy, convulsions, seizures, autoimmune disease, or immunodeficiency were excluded from the study. The research protocol was approved by the Scientific Review Committee and the Ethics Committee of Jiangsu Provincial Center for Disease Control and Prevention (approval number: JSJK2022-B014-02). Informed consent was obtained from each infant’s parents or legal guardians before participating.

Baseline information, including demographic data of the infants and their family members or caregivers, was collected through a face-to-face visit at enrollment, which also documented the previous disease history of the infants. Each infant was scheduled for regular follow-up visits for active surveillance of common ARIs after enrollment until reaching 18 months of age. Immunization records of the infants were obtained from the Vaccination Integrated Service Management Information System. The surveillance for common ARIs in the cohort is still ongoing.

Here, we report data from a follow-up of approximately 12 months, with the data cutoff on 30 April 2024.

### 2.2 Common ARI surveillance and specimen collection

Active common ARI surveillance has started with the first infant enrolled in the cohort. Researchers contacted the parents or caregivers once per every 2 weeks by telephone to follow the recent health status of the infants and any symptoms of common ARIs. Parents or caregivers were asked to contact the researchers when a suspected common ARI episode was noted in infants with at least one symptom of nasal congestion, sore throat, cough, expectoration, tachypnea, or panting as soon as possible after the onset of the symptoms (reporting within 24 h). Researchers arranged home visits within 48 h after receiving a reported episode of common ARIs and took throat swabs from the infants who were undergoing a common ARI. An episode of common ARI would be documented without sampling for a late-reported episode exceeding 24 h after the onset of symptoms or a case with no specimen available within 48 h after the report or before medicine administration. When parents reported more than one common ARI for an infant, a new episode of common ARIs was required to have an interval of at least 15 days after recovery from the previous one. Specimens were placed in a storage tube containing virus storage solution and stored in the refrigerator at −80°C before shipping for testing.

During the surveillance period, infants with ARIs who were hospitalized for treatment were documented, and the information associated with the respiratory pathogens were extracted from the discharge summaries after resolving.

### 2.3 Laboratory tests

#### 2.3.1 Targeted next-generation sequencing

The throat swab specimens were transported to Guangzhou Jinyu Medical Laboratory Center Co., LTD. (King Med Diagnostics, Guangzhou, China) on dry ice for testing. The tNGS sequencing was performed to identify the 107 common respiratory pathogens and specific 23S rRNA gene resistance mutations (A2063G, A2064G, A2067G, and A2617G) in *M. pneumoniae*, as well as A2047G in *B. pertussis* (Appendix 1: The table presents the detection targets identified by tNGS), using the Respiratory Pathogen Detection Kit (KS608-100HXD96, KingCreate, Guangzhou, China) following the instructions in the manual (18). Briefly, the throat swab specimens underwent nucleic acid extraction or purification using reagents (KS118-BYTQ-72, KingCreate, Guangzhou, China) on a nucleic acid extractor (KingFisher Flex, Thermo Fisher, United States), targeting the highly conserved regions of 107 respiratory pathogens with specific primers. Two rounds of PCR were performed. Library quality and quantity were assessed with Qsep100 and Qubit 4.0. Diluted, denatured libraries were sequenced on the Illumina MiniSeq. Sequencing data were filtered, aligned with a reference genome, and interpreted for pathogen detection.

#### 2.3.2 Multiplex polymerase chain reaction technique

The throat swab specimens with positive results of *S. pneumoniae* were further investigated in order to determine the specific serotypes by using mPCR. Following the manufacturer’s instructions, DNA was extracted from throat swab specimens using a nucleic acid extraction kit (T132, TIANLONG, China), followed by the PCR reaction. Subsequently, the amplified products were analyzed using the Qsep400 High-Throughput Nucleic Acid Protein Analysis system (C400100, Houzebio, Hangzhou, China).

### 2.4 Statistical analysis

The sample size was estimated using PASS software (2021, v21.0.3). To detect an incidence of 0.03 for certain respiratory pathogen-associated ARIs with a minimum reliability of 0.01, a total of 480 observations were able to provide at least 90% power at a 0.05 significance level, assuming an average of one episode per person-year. Given that approximately 40% of episodes of ARIs might be missed or not sampled for throat swabs during surveillance, the sample size was expanded to 800 individuals.

The overall incidence of common ARIs was calculated as the number of episodes of common ARIs divided by the total person-time of the cohort during surveillance. The monthly incidence rate is the number of common ARI episodes per month divided by the total number of people monitored in the cohort each month during the surveillance period. The pathogen composition ratio is calculated by taking the number of positive detections of a pathogen as the numerator and the total number of all pathogen-positive detections as the denominator. Positive rates are calculated by taking the number of positive cases for each pathogen as the numerator and the total number of ARI-tested cases as the denominator. For the coexisting ratio of pathogens “X” and “Y,” the numerator is the number of positive cases co-detected with both “X” and “Y,” while the denominator is the total number of common ARI-tested cases. Binary logistic regression models were used to examine factors that may affect the positivity of pathogens, including sex, age, seasons, infant disease history (including pneumonia, jaundice, COVID-19, and lactose intolerance), maternal mode of delivery, breastfeeding, maternal age, gravidity, parity, maternal education, maternal monthly income, the number of household members, presence of household members under 18 years old, as well as baseline pathogen carriages. We used the binary logistic regression models to analyze the factors influencing the positive detection rate of pathogens, primarily because it is well-suited for binary dependent variables (e.g., “positive/negative”) and allows for the quantification of the independent effects of multiple independent variables (e.g., sex, age, seasons, and infant disease history) on the outcome. Compared to the chi-square tests or univariate analysis, this model effectively controls for confounding factors and assesses the strength of associations between variables by calculating the odds ratio (OR).

The chi-square test or Fisher’s exact test was used for the inter-group comparison. All statistical analyses were performed using R version 4.3.3 (R Foundation for Statistical Computing, Vienna, Austria). A two-sided *p*-value of <0.05 was considered statistically significant. Figures were created with GraphPad Prism version 9.00 (GraphPad Software, San Diego, CA).

## 3 Results

### 3.1 Study participants

**Figure 1:**
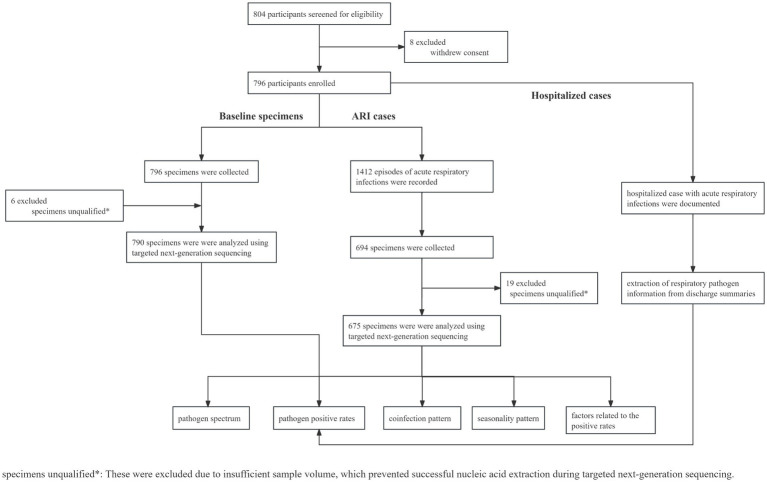
Flow diagram. The data cutoff was set on 30 April 2024.

Between 7 February and 17 April 2023, a total of 804 infants were invited to participate and screened for eligibility. Among them, 796 participants were eligible and included in the study cohort and followed up for approximately 12 months, covering the four seasons from spring to winter, with the data cutoff on 30 April 2024 (Figure 1). The median age of the infants was 71.0 days (interquartile range (IQR) 66.0–77.3 days), with 423 (53.1%) being male and 373 (46.9%) being female. The demographic data of infants and their family members or caregivers, including disease history, maternal mode of delivery, maternal feeding pattern, the number of household members, and the presence of household members under 18 years old, are detailed in Table 1.

**Table 1:** Demographic characteristics of infants enrolled (*N* = 796): Suqian, Jiangsu, China, February 2023–April 2024.

Characteristics	Total
Infant sex, *n* (%)	
Female	373 (46.9)
Male	423 (53.1)
Infant age, median (IQR), days	71.0 (66.0, 77.3)
Infant region, *n* (%)	
Sucheng	198 (24.9)
Suyu	296 (37.2)
Siyang	302 (37.9)
Infant height, median (IQR), cm	60.0 (59.0, 62.0)
Infant weight, median (IQR), kg	6.3 (5.8, 6.8)
Infant gestational age, median (IQR), weeks	39.0 (38.0, 40.0)
Infant birth length, median (IQR), cm	50.0 (50.0, 50.0)
Infant birth weight, median (IQR), kg	3.4 (3.2, 3.7)
Infant disease history*, *n* (%)	
Yes	22 (2.8)
No	774 (97.2)
Maternal mode of delivery, *n* (%)	
Full-term vaginal delivery	399 (50.1)
Cesarean delivery	397 (49.9)
Maternal feeding pattern, *n* (%)	
Breastfeeding	701 (88.1)
Not breastfeeding after birth	80 (10.1)
Mixed feeding	15 (1.9)
Maternal age, *n* (%), years	
<35	687 (86.3)
≥35	109 (13.7)
Gravidity, *n* (%)	
1	267 (33.5)
2	263 (33.0)
>2	266 (33.4)
Parity, *n* (%)	
1	329 (41.3)
2	363 (45.6)
>2	104 (13.1)
Maternal education, *n* (%)	
Middle school or below	210 (26.4)
High school or vocational school	141 (17.7)
Junior college	223 (28.0)
Bachelor degree or above	222 (27.9)
Maternal monthly income, *n* (%), CNY	
<1, 000	329 (41.3)
1, 000–6, 999	370 (46.5)
≥7, 000	97 (12.2)
No. of household members, *n* (%)	
≤ 4	584 (73.4)
>4	212 (26.6)
With household members <18 years of age, *n* (%)	
Yes	437 (54.9)
No	359 (45.1)
Monthly household income, *n* (%), CNY	
<10, 000	237 (29.8)
≥10, 000	559 (70.2)

Data are n (%) unless otherwise indicated. Percentages may not total 100 because of rounding. *Infant disease history included pneumonia, jaundice, COVID-19, and lactose intolerance.

### 3.2 Common ARI incidence

**Figure 2:**
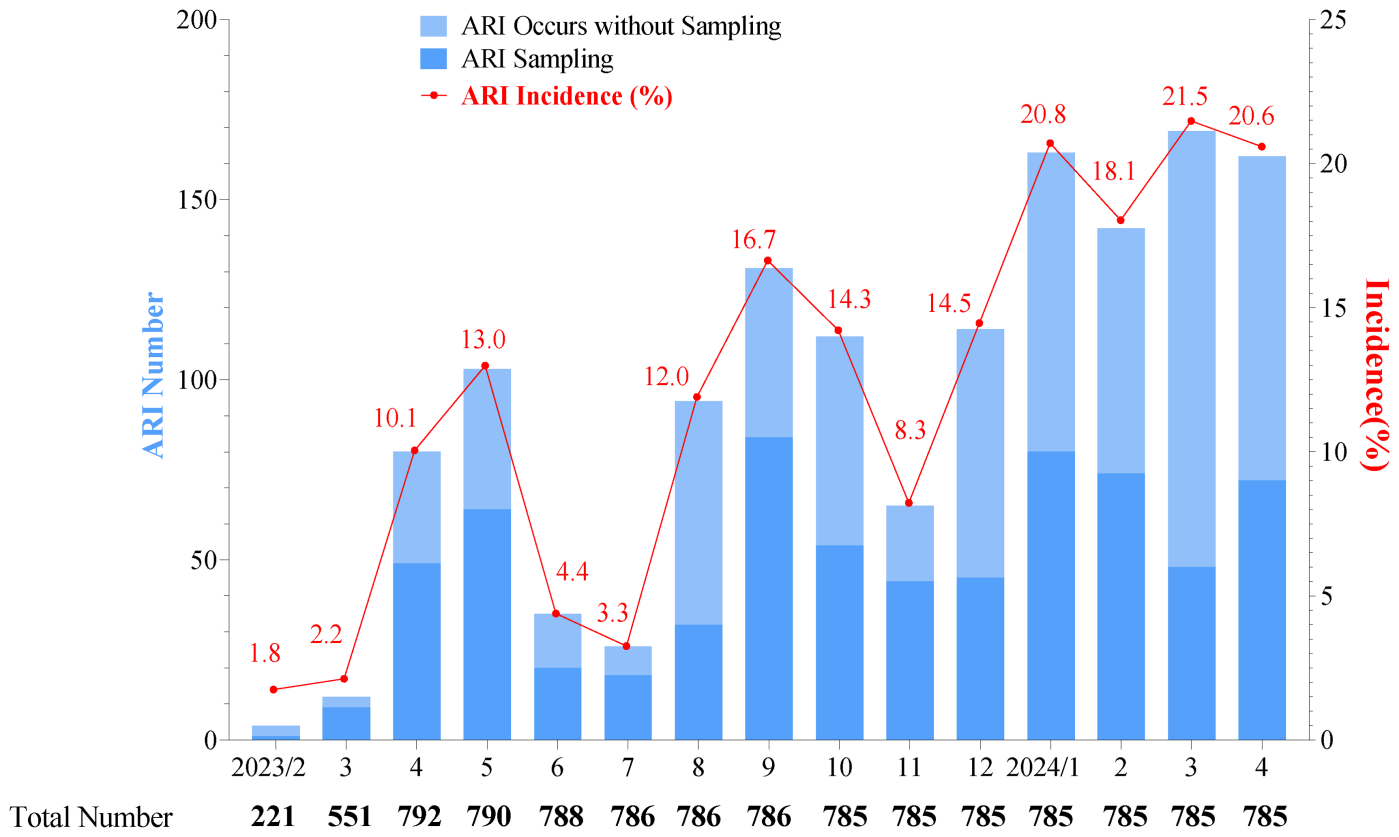
Monthly distribution of acute respiratory infection cases (bars) and incidence rate (line and spot) in a cohort surveillance from February 2023 to April 2024. The total number represents the participants in our cohort, and as monitoring continues, some participants may exit. “ARI sampling” refers to cases where a participant experiences ARIs and completes the sample collection process. “ARI occurs without sampling” refers to cases with symptoms identified as an episode of ARIs, but with no sample available for pathogen detection. As there was only one specimen in February 2023, this specimen was included in the March 2023 analysis. The monthly incidence of ARIs was low from February to March 2023, which might be attributed to the ongoing enrollment of the cohort.

From 7 February 2023 to 30 April 2024, a total of 1,412 common ARI cases among infants were documented, with an average of 164.5 episodes per 100 person-years. The highest monthly incidence rates for common ARIs were observed between January and April 2024, ranging from 18.1 to 21.5%. This was followed by two smaller peaks of common ARIs in May and September 2023, with monthly incidence rates of 13.0 and 16.7%, respectively (Figure 2). Among all the clinical symptoms of common ARI episodes recorded, nasal congestion (35.9%) was the most commonly reported symptom, followed by cough (23.0%), fever (1.5%), expectoration (0.4%), and other symptoms (0.3%). Additionally, 39.0% of the common ARI (263/675) cases presented with two or more clinical symptoms, with nasal congestion and cough being the most common combination (19.9%, 134/675), followed by fever and nasal congestion (4.2%, 28/675) and fever and cough (4.0%, 27/675) (Appendix 2).

### 3.3 Pathogen positive rates

**Figure 3:**
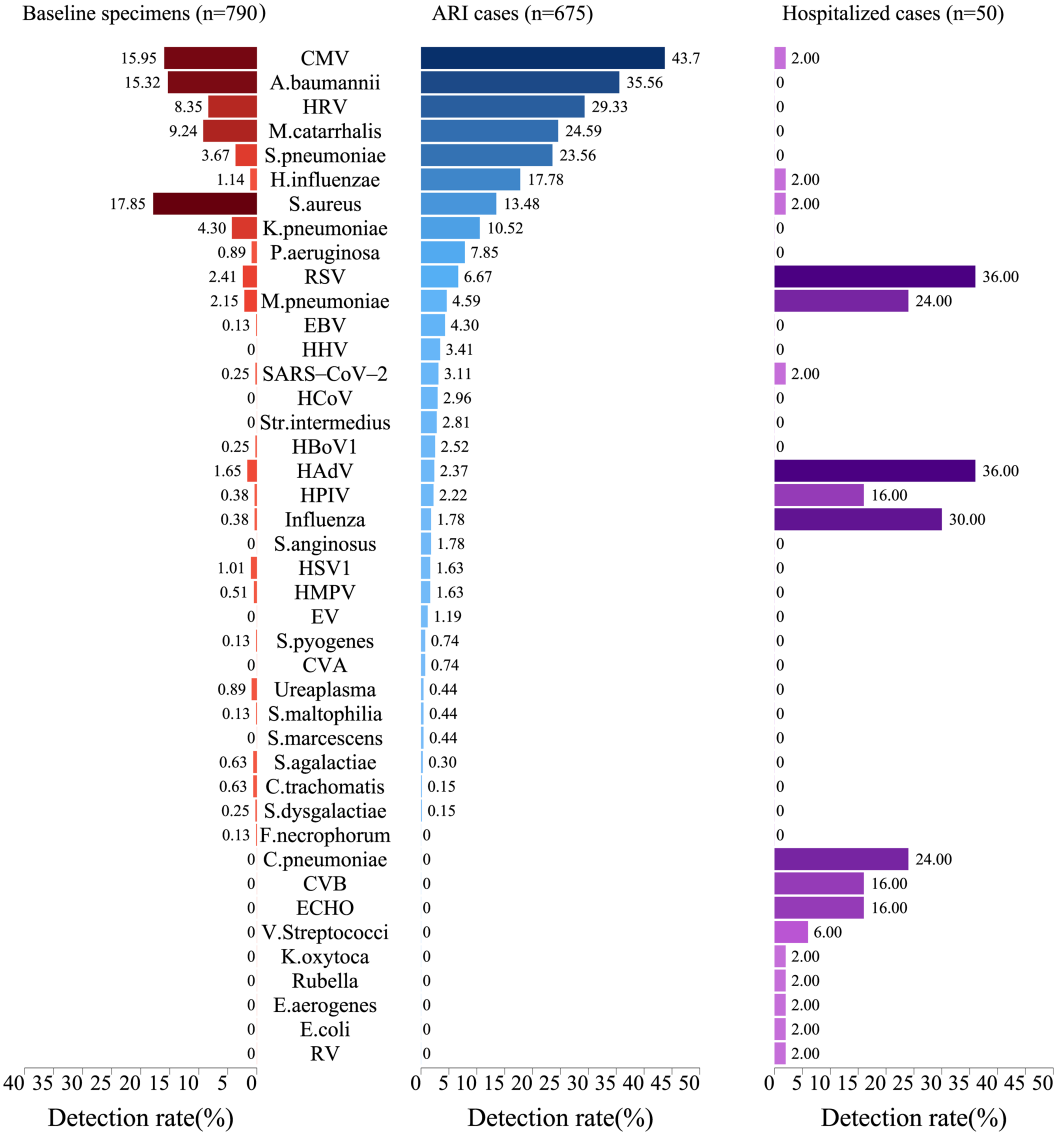
Positive rates of pathogens in infants at baseline, during acute respiratory infections, and in hospitalized cases. The width of the colored bars and the number beside indicate the positive rate of each pathogen. Positive rate was calculated by taking the number of positive cases for each pathogen as the numerator and the total number of specimens tested as the denominator.

A total of 796 baseline specimens were collected from infants at enrollment, and 694 specimens were collected from 409 infants during common ARIs, while 6 baseline specimens and 19 common ARI specimens were excluded due to an insufficient amount of nucleic acid extracted for testing. At enrollment, 278 healthy infants (35.2%) were positive with at least one pathogen as baseline carriers. The top five pathogens identified at baseline were *Staphylococcus aureus (S. aureus)* (17.9%), Cytomegalovirus (CMV) (16.0%), *Acinetobacter baumannii (A. baumannii)* (15.3%), *Moraxella catarrhalis (M. catarrhalis)* (9.2%), and HRV (8.4%) (Figure 3). For the specimens of common ARIs, 636 (94.2%) specimens were positive for at least one respiratory pathogen. The highest pathogen detection rate, i.e., 96.2% [150/156], was observed when infants were aged 7–9 months, 95.0% (341/359) when infants were aged 10–16 months, 93.1% (95/102) when infants were aged 4–6 months, and 86.2% (50/58) when infants were aged 2–3 months. No significant difference in the positive rate was found between boys (94.1%) and girls (94.4%) (Appendix 3: The table shows the positive detection rates of pathogens in common infant cases).

**Figure 4:**
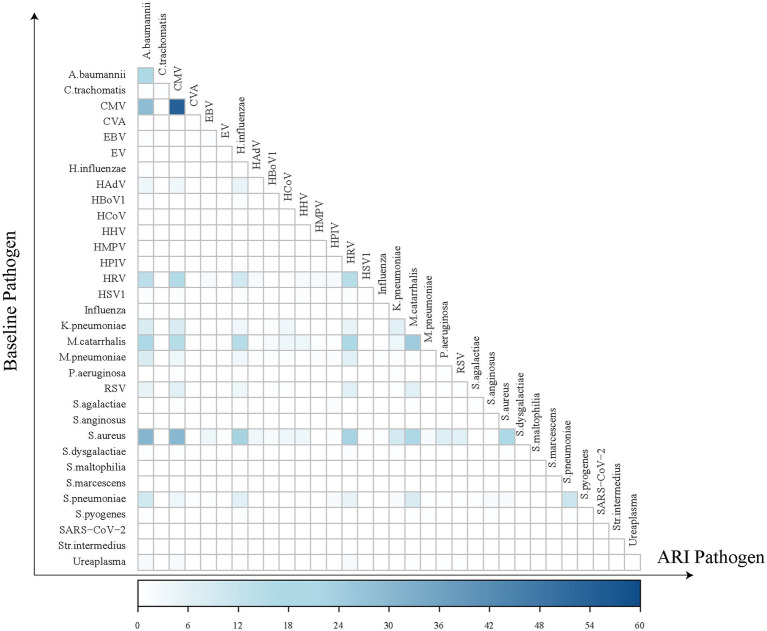
Heatmap of pathogen co-detection frequency between baseline and acute respiratory infection. The horizontal axis represents pathogens detected during ARI episodes, while the vertical axis represents those identified at baseline. Each square in the heatmap indicates the frequency of individuals who tested positive for a specific pathogen at baseline and either the same or a different pathogen during an ARI episode. The darker the color of the square, the greater the number of individuals with the co-detection of these pathogens at both baseline and during ARI episodes.

Regarding the positive detection rates of pathogens in common ARIs, CMV exhibited the highest positive rate at 43.7%, followed by *A. baumannii* at 35.6%, HRV at 29.3%, *M. catarrhalis* at 24.6%, and *S. pneumoniae* at 23.6% (Figure 3). A heatmap analysis of whether the presence of certain pathogens at baseline affected their detection during subsequent common ARI episodes indicated that CMV, *M. catarrhalis*, *A. baumannii*, HRV, *S. aureus, and S. pneumoniae* were more likely to be detected during common ARIs when they were already present at baseline, suggesting that these pathogens may be classified as long-term colonizers (Figure 4).

We recorded 57 hospitalized episodes of ARIs. Among them, 50 had specimens for measurement, and 31 (62.0%) were tested positive for at least 1 respiratory pathogen (Appendix 4: The table presents the positive rate of any pathogen in hospitalized infant cases.). The pathogen detection results from the hospitalized episodes of ARIs differed notably from those of non-hospitalized ARI cases, highlighting a distinct pathogen profile between the two groups. Among hospitalized ARI episodes, HAdV and RSV demonstrated the highest positive detection rates at 36.0%, followed by influenza at 30.0%, and both *M. pneumoniae and Chlamydia pneumoniae* (*C. pneumoniae*) at 24.0% (Figure 3). Sex-specific analysis revealed significantly higher positive detection rates in girls compared to those in boys in terms of influenza A and human parainfluenza virus (HPIV) for the hospitalized episodes of ARIs (Appendix 5: The table displays the positive detection rates of pathogens in hospitalized infant cases, categorized by gender and age group.).

### 3.4 Pathogen spectrum

**Figure 5:**
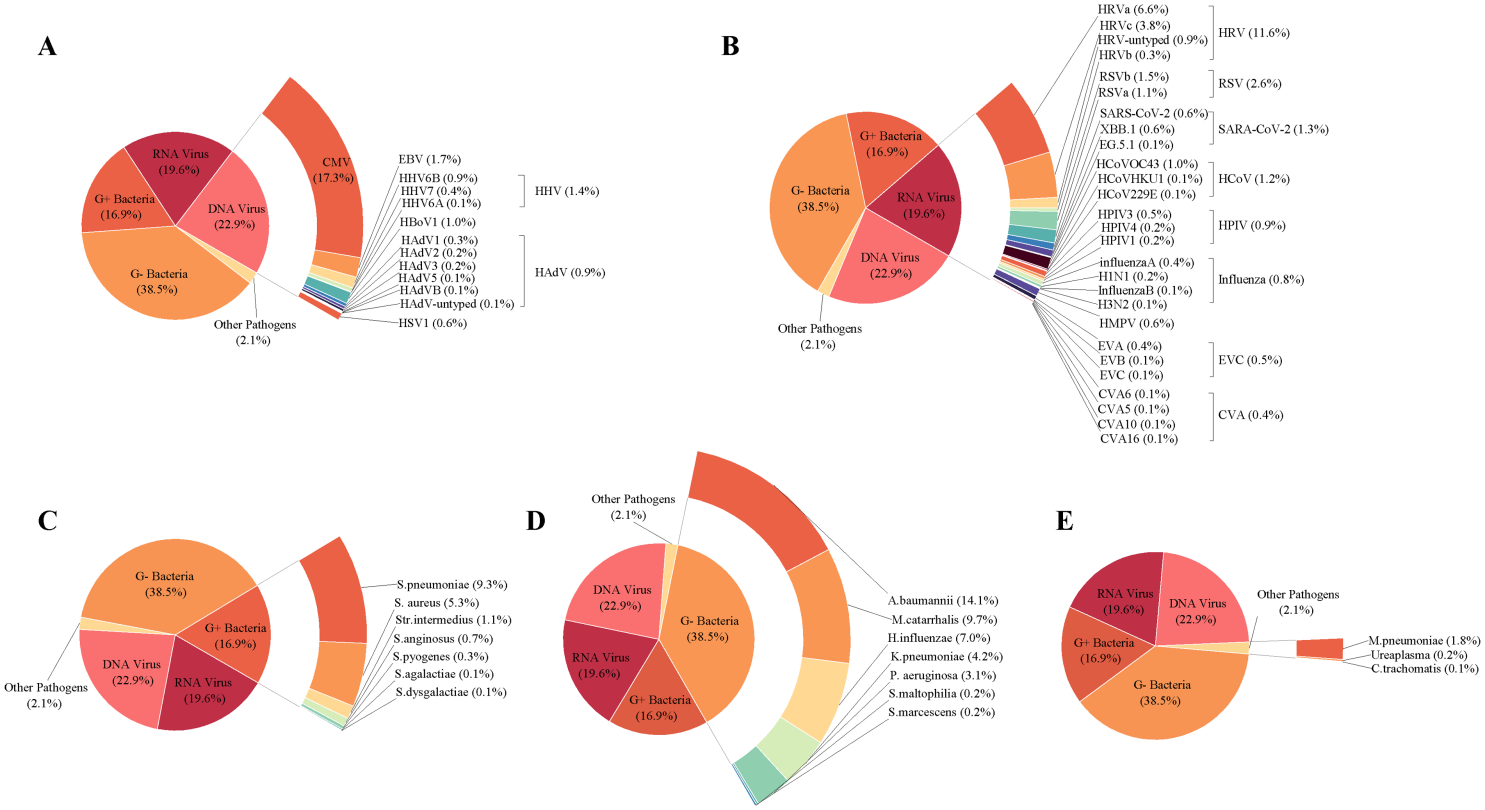
Composition of pathogens detected in 675 infants with acute respiratory infections. The composition of DNA viral pathogen **(A)**, RNA viral pathogen **(B)**, Gram-positive bacteria **(C)**, Gram-negative bacteria **(D)**, and other pathogens **(E)** was detected in 675 infants with acute respiratory infections. The overall viral composition of 675 ARI cases had all 107 pathogens tested. The fragment size of colored bars and the number behind indicate the proportion of each pathogen, calculated by its positive number used as the numerator and the total positive number of all pathogens used as the denominator. “Other pathogens” refer to pathogens besides viruses and bacteria.

The pathogen spectrum of 675 specimens for common ARIs presented a total of 1,706 pathogens. CMV was the most frequently identified DNA viral pathogen, constituting 17.3% of all pathogens identified in common ARIs. HRV stood out as the most frequently detected RNA viral pathogen, representing 11.6% of all detected pathogens. Further genotyping analysis of HRV revealed the predominant presence of HRV-A, accounting for 6.6% of all detected pathogens, in contrast to HRV-C (3.8%), HRV-untyped (0.9%), and HRV-B (0.3%). RSV-B and RSV-A comprised 1.5 and 1.1% of all detected pathogens from common ARIs, respectively. Among the genotyped SARS-CoV-2 (1.3%), Omicron-XBB.1 was the most prevalent SARS-CoV-2 variant at 0.6%, followed by Omicron-EG.5.1 at 0.1%. In terms of Gram-positive bacteria, *S. pneumoniae* emerged as the most prevalent, accounting for 9.3% of all detected pathogens. *A. baumannii* was the most frequently identified Gram-negative bacteria, constituting 14.1% of positive specimens among all detected pathogens. Among other pathogens besides viruses and bacteria, *M. pneumoniae* was the most commonly detected, accounting for 1.8% of all pathogens identified (Figure 5).

In addition, macrolide resistance mutation A2063G was detected in 14 out of all samples that tested positive for *M. pneumoniae*. Moreover, 155 out of 159 specimens positive for *S. pneumoniae* were subjected to further analysis using mPCR for serotyping (4 were excluded for insufficient volume). Of them, 65 were identified and categorized into 14 serotypes of *S. pneumoniae*. The top three frequently identified serotypes were 15A/15F (25 strains, 38.5%), 10F/10C/33C (15 strains, 23.1%), and 10A (9 strains, 13.8%) (Appendix 6: The table summarizes the serotyping results for *S. pneumoniae*).

### 3.5 Coinfection pattern

Coexistence with two or more pathogens was present in 497 cases (73.6%), among which 312 cases (46.2%) involved three or more coexisting pathogens. Viral–bacterial coexistence remained the predominant type across all age groups (Appendix 7: The table presents the co-detection rates of pathogens in common infant ARI cases across various age groups.). CMV, HRV, *A. baumannii*, and *M. catarrhalis* were the top four pathogens prone to coexisting with other pathogens. Among these combinations, CMV-*A. baumannii* coexistence exhibited the highest positive rate at 15.4%, followed by CMV-HRV (12.1%), CMV-*S. pneumoniae* (11.9%), and CMV-*M. catarrhalis* (11.3%) (Appendices 8, 9).

### 3.6 Seasonal patterns

**Figure 6:**
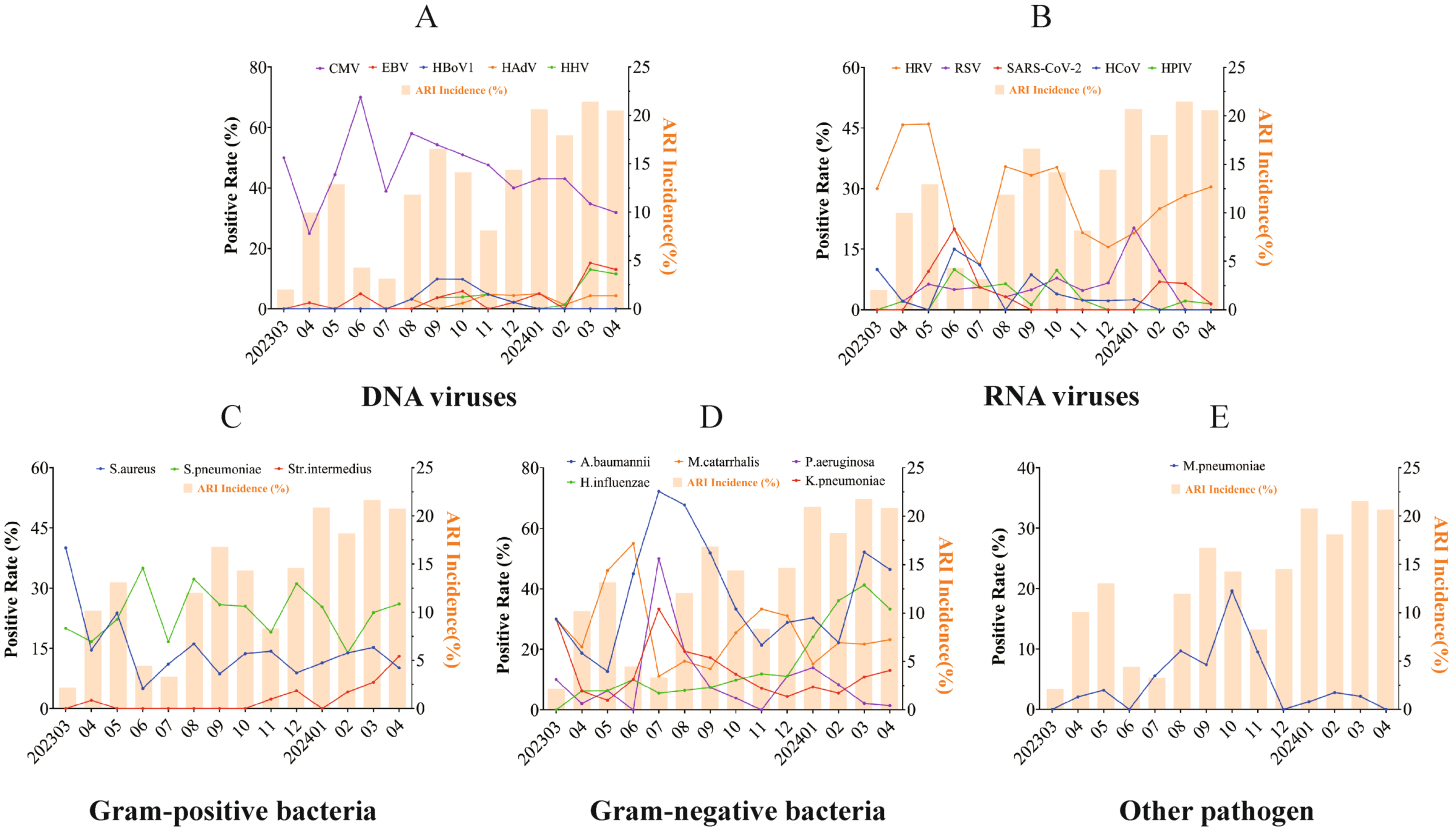
Seasonality pattern of respiratory pathogens in acute respiratory infections. Seasonal patterns of respiratory pathogens in acute respiratory infections are shown for DNA viral pathogens **(A)**, RNA viral pathogens **(B)**, Gram-positive bacteria **(C)**, Gram-negative bacteria **(D)**, and other pathogens **(E)**. Seasonal analysis of pathogen-positive detection rates was conducted for pathogens with an overall positive rate exceeding 2.0%. Each line and spot indicate the monthly average positive rate of the corresponding pathogen. The bars represent the seasonal distribution of overall respiratory infections, while the lines and dots illustrate the seasonal prevalence trends for different pathogens.

Seasonal patterns of pathogens were presented for the pathogens of common ARIs with an overall positive rate exceeding 2.0%. Among DNA viruses, CMV was prevalent consistently throughout the year, with higher positive detection rates observed during the summer and autumn. Epstein–Barr virus (EBV) and human herpes virus (HHV) exhibited a marked increase in March 2024, reaching their epidemic peak in the spring (March–April 2024), while human bocavirus 1 (HBoV1) peaked in the autumn. In terms of RNA viruses, HRV displayed high positive detection rates year-round, with higher rates observed during the spring. RSV and SARS-CoV-2 exhibited distinct seasonal patterns, with RSV peaking during the colder months (December 2023 to February 2024), while SARS-CoV-2 reached its epidemic peak in the warmer months (June 2024). Human coronavirus (HCoV) and HPIV did not show distinct seasonality. Similar to a Gram-positive bacteria, *S. pneumoniae* is prevalent year-round. *S. aureus* exhibited a peak in March 2023, with a relatively consistent detection rate in the remaining months. *Streptococcus intermedius (S. intermedius)* was infrequently detected or even absent in most months but experienced an epidemic peak between March and April 2024. Among Gram-negative bacteria, *M. catarrhalis* was consistently detected throughout the year, with a peak in June, while *A. baumannii*, *Klebsiella pneumoniae* (*K. pneumoniae*), *Pseudomonas aeruginosa* (*P. aeruginosa*), and predominantly circulated in the warm seasons. Additionally, *K. pneumoniae* and *H. influenzae* were also found to be prevalent in early spring. Finally, *M. pneumoniae* primarily circulated during the warm seasons (Figure 6).

### 3.7 Factors related to the positive rate

According to multivariate logistic regression analysis, we found that, when CMV, *S. pneumoniae*, *M. catarrhalis,* and *S. aureus* were present at baseline, the risk of positive detection during common ARIs significantly increased, with ORs of 5.96 (95% CI: 3.37–11.01) (*p* < 0.0001), 3.28 (95% CI: 1.37–7.91) (*p* = 0.0074), 2.86 (95% CI: 1.61–5.12) (*p* = 0.00036), and 2.31 (95% CI: 1.29–4.04) (*p* = 0.0039), respectively. In addition, a significant increase in the incidence of CMV was associated with breastfeeding, with an OR of 20.88 (95% CI: 6.03–132.36) (*p* < 0.0001). In contrast, living with household members under 18 years of age decreased the risk of CMV positivity with an OR of 0.57 (95% CI: 0.37–0.89) (*p* = 0.013). Positive detections of *S. pneumoniae* or *M. catarrhalis* increased when participants lived with other children under the age of 18 in the family, with ORs of 4.64 (95% CI: 2.77–7.69) (*p* < 0.0001) and 3.02 (95% CI: 1.84–5.03) (*p* < 0.0001), respectively. An increased incidence of *M. pneumoniae* was associated with infants who had a history of diseases with an OR of 7.82 (95% CI: 1.62–34.38) (*p* = 0.0072) (Appendices 10–16).

### 3.8 Vaccination records

The vaccination records of the infants were collected up to 30 April 2024 (Appendix 17). Among all common ARI cases, 18.4% (14/76) of those who completed three doses of the 13-valent pneumococcal vaccine tested positive for *S. pneumoniae*, whereas 29.7% (99/333) of those who did not complete all three doses tested positive for *S. pneumoniae*. Among 790 infants who completed three doses of the DTaP-Hib-IPV vaccine, 100 individuals tested positive for *H. influenzae*, resulting in a positive rate of 12.7%. However, among these common ARIs, only eight individuals tested positive solely for *H. influenzae*. Additionally, none of the common ARIs were positive for *B. pertussis*. Subsequent to the initial administration of the IIV4 to 158 individuals, one infant tested positive for influenza A virus, with a positive rate of 0.6% after influenza vaccination.

## 4 Discussion

Here, we presented an up-to-date picture of respiratory infection pathogens affecting infants and young children with episodes of common ARIs, monitored from 7 February 2023 to 30 April 2024.

The occurrence of common ARIs among infants varied between seasons, with an overall incidence of 164.5 episodes per 100 person-years. This overall incidence is slightly lower than the reported age-standardized incidence rates of upper respiratory infections in China in 2019, before the COVID-19 pandemic, which were estimated to be 179 episodes per 100 person-years (19). However, incidence rates in the peak period for common ARIs occurred between January and April 2024 in our study and were notably higher than the annual average incidence, with monthly incidence rates ranging from 18.1 to 21.5%. The most common type of coexisting pathogens was viral-bacterial coexistence. More virus–bacterial coexistence is potentially attributed to respiratory viruses impeding the growth of other viruses through resource competition, immune response modulation, or viral protein interactions (20). In contrast, viral pathogen infections can damage airways, enhance bacterial adhesion, and disrupt the host immune system, thereby promoting bacterial growth. Bacterial infection, in turn, may facilitate viral infection by enhancing bacterial adhesion and altering viral transmission, thereby contributing to immune evasion (21). These bidirectional effects together contribute to the pathogenesis of viral-bacterial co-infection.

In our study, CMV exhibited the highest positive detection rate (43.7%) among infants and young children with common ARIs, followed by *A. baumannii*, HRV, *M. catarrhalis*, and *S. pneumoniae*, with positive rates ranging approximately 23.6 ~ 35.6%, accounting for the majority of respiratory pathogens detected. We explored the association between the patterns and distributions of respiratory pathogen carriage in infants at baseline and the respiratory pathogens among those with common ARIs. CMV, *A. baumannii, M. catarrhalis,* and HRV were the most commonly detected pathogens at baseline, acting as carriers among these infants. The presence of CMV, *S. pneumoniae*, *M. catarrhalis,* and *S. aureus* at baseline significantly increased the risk of positive detection during ARIs by 2.31 ~ 5.96-fold, respectively.

In contrast, the respiratory pathogens observed in those with common ARIs and those with ARI-associated hospitalization were significantly different. Although RSV ranked third and showed 6.7% incidence rates among viral pathogens in infants with common ARIs, it contributed more than one-third (36.0%) of hospitalized children with ARIs, highlighting its significant impact on public health and the potential for substantial disease burden (22). Besides RSV, HAdV (36.0%), Influenza (30.0%), *M. pneumoniae* (24.0%) and *C. pneumoniae* (24.0%) also exhibited high positive detection rates in hospitalized cases in our study, while their positive detection rates were notably low (2.4, 1.8, 4.6, and 0.0%, respectively) in infants and children with common ARIs. This finding indicates an obvious difference in pathogen composition between hospitalized ARI cases and common ARI cases. The pathogens identified in hospitalized cases are associated with more severe symptoms that necessitate hospitalization, suggesting that they may possess a higher pathogenic potential.

We also found that the positive detection rates of some bacterial pathogens in common ARI cases are high, with 42.9% (6/14) of them exceeding 10%. However, in hospitalized cases, the positive detection rate for bacterial pathogens is generally minimal. This discrepancy may be because most of these bacteria are opportunistic pathogens, detected in common ARI cases, possibly due to being carriers rather than being the actual cause of the illness (23). Our study also confirmed that the bacterial pathogens (*A. baumannii*, *M. catarrhalis*, *S. pneumoniae*, and *S. aureus*) with higher positive detection rates were more likely to be detected during common ARIs when they were already present at baseline, suggesting that these pathogens may be classified as long-term colonizers.

In this study, we conducted serotyping of *S. pneumoniae*, which makes up for this aspect that is missing in many birth cohorts. The predominant serotypes identified in our study were 15A/15F and 10F/10C/33C, which differed from those commonly observed in children with acute respiratory infections in other research studies (24, 25). This variation could be attributed to the fact that the ARI cases in our study involved children with mild symptoms, unlike the focus on hospitalized children in previous research. Our research contributes to the understanding of *S. pneumoniae* serotypes in non-hospitalized cases of ARIs among infants and young children.

In addition, *H. influenzae* is a conditionally pathogenic bacterium frequently isolated from respiratory specimens in children (26). We recorded 100 infants with common ARI episodes positive for *H. influenzae* after completing the primary immunization of DTaP-Hib-IPV in this study, but only 8 of them were positive solely for *H. influenzae*, indicating that the presence of *H. influenzae* may be a result of coinfection or colonization. Nevertheless, the relatively high detection rate of *H. influenzae* even after vaccination indicates that it is worthwhile to pay attention to the disease burden associated with *H. influenzae* in infants and young children, particularly those who have not been vaccinated against *H. influenzae*.

Although these pathogens are typically regarded as colonizers, their significance in long-term immune monitoring should not be underestimated. CMV, for instance, can persist in the host for extended periods, potentially leading to immune escape or suppression, which, in turn, affects the immune response to other pathogens. Studies have linked CMV infection to immune aging, chronic diseases, and autoimmune conditions (27). Therefore, continuous monitoring of CMV colonization in children is essential for developing effective immune strategies and preventive measures. Similarly, *S. pneumoniae* commonly colonizes the upper respiratory tract in children and is closely associated with the onset of acute infections and the recurrence of chronic diseases (28). Long-term surveillance of its colonization is critical for assessing vaccine efficacy and informing immune strategies targeting specific serotypes. Furthermore, *M. catarrhalis*, which also colonizes the upper respiratory tract, is implicated in recurrent respiratory infections and increases the risk of otitis media, pneumonia, and other related infections (29). Ongoing monitoring of its colonization is vital for understanding its immunological role. In conclusion, dynamic surveillance of certain colonizing pathogens can offer valuable insights and contribute to the development of precise interventions for pediatric respiratory infections.

We also explored the factors that could affect the positivity of respiratory pathogens among infants and young children in this study. Breastfeeding was identified as an important factor increasing the risk of CMV pathogen detection in infants, which is consistent with the fact that CMV can be transmitted to infants through breast milk (30). Additionally, living with other household members under 18 years of age was associated with an increased risk of testing positive for *S. pneumoniae* and *M. catarrhalis* but a decreased risk of testing positive for CMV. Although the exact reason for this is unclear, one possible explanation is that older children who have already been infected with CMV may provide a level of “herd immunity” in the family, protecting the infants or young children from exposure to CMV (31). Nevertheless, a comprehensive analysis of specific familial contexts, hygiene practices, cultural backgrounds, and research design factors is warranted.

Only two publications studied respiratory infection pathogens during the 2023–2024 period in China, and only one specifically focused on young children. These studies highlighted the persistent prevalence of *M. pneumoniae* and an increasing trend in influenza infections (32, 33). However, both studies were conducted during the flu season (September 2023 to March 2024), while our study’s positive detection rate of the influenza virus spans the entire year. Additionally, the previous studies primarily focused on severe patients who sought hospital care or were hospitalized, whereas our study examines individuals with mild acute respiratory infections, which may result in differences in the pathogen composition. Moreover, our study also included hospitalized ARI cases, where the pathogen detection results show a higher positive rate of influenza, second only to HAdV and RSV. These data stress the importance of understanding the complexity of respiratory infection pathogens affecting infants and young children.

## 5 Advantages

In this study, we reported a prospective cohort study of surveillance of ARIs among infants in China from 2023 to 2024. Given the critical role of ongoing ARI surveillance in identifying the prevalence of respiratory pathogens, assessing risk factors and health disparities, and informing prevention policies, our study provides valuable insights into the epidemiology of ARIs in this population.

## 6 Limitations

Nevertheless, our study has some limitations. One limitation of this study is the challenge of establishing causality based solely on the positive detection of pathogens, particularly for bacterial pathogens, as a positive result may indicate colonization rather than infection. Furthermore, these findings are based on epidemiological patterns observed in a restricted sample of infants with mild acute respiratory infections across three neighboring locations, which may not fully represent other infant populations or more severe infection cases. For instance, variations in healthcare access, environmental factors, and socioeconomic status in different regions can influence the incidence and severity of respiratory infections among infants. Therefore, caution should be exercised when extrapolating these results to other groups or settings, as they may not adequately represent the complexities of respiratory infections in infants on a larger scale. Additionally, our research site is in eastern coastal China, characterized by a warm-temperate sub-humid monsoon climate, which could lead to variations in pathogen distribution compared to tropical or frigid climate zones. Underreporting can occur during both monitoring and sampling, potentially introducing uncertainty into the study’s findings and leading to an underestimation of common ARI incidence. However, such instances of underreporting during monitoring and sampling are expected to be random and unordered and do not significantly affect our results. Although this study utilized a cohort design, potential selection bias remains, particularly due to participant dropout during the follow-up period. Furthermore, the questionnaire did not account for all possible confounding factors, and these were not adjusted for in the analysis, leading to statistical limitations that may affect the generalizability of the findings.

## 7 Conclusion

This study identified the dominant pathogens of pediatric ARIs as CMV, *A. baumannii*, and HRV for common ARIs and HAdV and RSV for hospitalized ARIs. Among these pathogens, CMV is more prevalent in the summer and autumn, HRV peaks in the spring, and *S. pneumoniae* circulates year-round. Therefore, vaccination or preventive measures for pathogens such as CMV, *S. pneumoniae*, and *M. catarrhalis* should be considered prior to their peak seasons, while stronger preventive measures for seasonal viruses such as influenza and RSV are also recommended.

In conclusion, our analysis provides a comprehensive overview of the respiratory pathogen spectrum, as well as the patterns and trends of ARIs, offering valuable data for understanding the complexity of pathogen detection and co-detection during the respiratory illness upsurge in infants and children from 2023 to 2024. This study underscores the importance of ongoing surveillance of ARIs and the exploration of pathogen dynamics to inform respiratory disease prevention and child health policy formulation. Future research should focus on multi-center validation to enhance the applicability and accuracy of these findings across diverse regions. Furthermore, integrating these surveillance data into public health strategies is crucial for improving the prevention and management of ARIs in children.

## Data Availability

The raw data supporting the conclusions of this article will be made available by the authors, without undue reservation.
