# Supplementary material for: Respiratory pathogens in infants and young children with acute respiratory illness: a prospective cohort study, China, 2023–2024
Source: Front Public Health. 2025 Aug 4;13:1548190. doi: 10.3389/fpubh.2025.1548190 (PMC12358494; doi:10.3389/fpubh.2025.1548190)
Supplement: Supplementary file 1 [file Data_Sheet_1.docx]

Supplementary Material

Appendix

Content

[Appendix 1. Detection targets in multiplex polymerase chain reaction (mPCR) and targeted next-generation sequencing (tNGS) analysis. 1](#_Toc14368)

[Appendix 2. Proportional venn diagram of symptom overlap seen of acute respiratory infections among infant cases (n=675). 3](#_Toc24251)

[Appendix 3. Positive rate of any pathogen in 675 infant case presenting with acute respiratory infections during February, 2023 to April, 2024. 4](#_Toc29160)

[Appendix 4. Positive rate of any pathogen in 50 hospitalized infant cases. 5](#_Toc31764)

[Appendix 5. Positive rate of respiratory pathogens among 50 Hospitalized infant cases by gender, age group. 6](#_Toc18530)

[Appendix 6. Summary of serotyping results of](#_Toc16692) *[S. pneumoniae](#_Toc16692)* [[n (%)]. 7](#_Toc16692)

[Appendix 7. Comparison of co-detection rates among infant ARI cases of different age groups by different types. 8](#_Toc8958)

[Appendix 8. Coexistence patterns of respiratory pathogens in 675 infant cases of acute respiratory infections. 9](#_Toc10382)

[Appendix 9. Coexistence pattern of respiratory pathogens among ARI cases. 10](#_Toc10241)

[Appendix 10. Factors related to the positive rate of CMV in infants among infant ARI cases 11](#_Toc1652)

[Appendix 11. Factors related to the positive rate of HRV in infants among infant ARI cases 12](#_Toc27944)

[Appendix 12. Factors related to the positive rate of](#_Toc10643) *[S. pneumoniae](#_Toc10643)* [in infants among infant ARI cases 13](#_Toc10643)

[Appendix 13. Factors related to the positive rate of](#_Toc32430) *[S. Aureus](#_Toc32430)* [in infants among infant ARI cases 14](#_Toc32430)

[Appendix 14. Factors related to the positive rate of](#_Toc3190) *[A. baumannii](#_Toc3190)* [in infants among infant ARI cases 15](#_Toc3190)

[Appendix 15. Factors related to the positive rate of](#_Toc30534) *[M. catarrhalis](#_Toc30534)* [in infants among infant ARI cases 16](#_Toc30534)

[Appendix 16. Factors related to the positive rate of](#_Toc2061) *[M. pneumoniae](#_Toc2061)* [in infants among infant ARI cases 17](#_Toc2061)

[Appendix 17. Evolution of vaccine coverage Among participants in the cohort over monitoring period. 18](#_Toc30973)

# Appendix 1. Detection targets in multiplex polymerase chain reaction (mPCR) and targeted next-generation sequencing (tNGS) analysis.

| Category | | Detection Targets | | |
| --- | --- | --- | --- | --- |
| Virus | DNA virus | Human adenovirus, HAdV | Human adenovirus 8, HAdV-8 | Herpes simplex virus 1, HSV-1 |
|  |  | Human adenovirus A, HAdV-A | Human adenovirus 12, HAdV-12 | Herpes simplex virus 2, HSV-2 |
|  |  | Human adenovirus B, HAdV-B | Human adenovirus 18, HAdV-18 | Varicella zoster virus, VZV |
|  |  | Human adenovirus C, HAdV-C | Human adenovirus 21, HAdV-21 | Epstein-Barr virus, EBV |
|  |  | Human adenovirus D, HAdV-D | Human adenovirus 24, HAdV-24 | Cytomegalovirus, CMV |
|  |  | Human adenovirus E, HAdV-E | Human adenovirus 27, HAdV-27 | Human herpes virus-6, HHV6 |
|  |  | Human adenovirus 1, HAdV-1 | Human adenovirus 28, HAdV-28 | Human herpes virus-6A, HHV6A |
|  |  | Human adenovirus 2, HAdV-2 | Human adenovirus 30, HAdV-30 | Human herpes virus-6B, HHV6B |
|  |  | Human adenovirus 3, HAdV-3 | Human adenovirus 31, HAdV-31 | Human herpes virus-7, HHV7 |
|  |  | Human adenovirus 4, HAdV-4 | Human adenovirus 34, HAdV-34 | Human bocavirus 1, HBoV1 |
|  |  | Human adenovirus 5, HAdV-5 | Human adenovirus 38, HAdV-38 | Human Parvovirus B19, HPVB19 |
|  |  | Human adenovirus 7, HAdV-7 | Human adenovirus 55, HAdV-55 |  |
|  | RNA virus | Enterovirus, EV | Human Coronavirus NL63, HCoV-NL63 | Influenza A virus H1N12009 |
|  |  | Enterovirus A, EV-A | Human Coronavirus OC43, HCoV-OC43 | Influenza B virus, IBV |
|  |  | Enterovirus B, EV-B | Human metapneumovirus, HMPV | Influenza C virus, ICV |
|  |  | Enterovirus C, EV-C | Human respiratory syncytial virus A, RSV-A | Measles virus, MV |
|  |  | Enterovirus D, EV-D | Human respiratory syncytial virus B, RSV-B | Mumps virus, MuV |
|  |  | Enterovirus A71, EVA71 | Human parainfluenza virus 1, HPIV-1 | Rhinovirus, HRV |
|  |  | Enterovirus D68, EVD68 | Human parainfluenza virus 2, HPIV-2 | Rhinovirus A, HRV-A |
|  |  | Coxsackievirus A5, CVA5 | Human parainfluenza virus 3, HPIV-3 | Rhinovirus B, HRV-B |
|  |  | Coxsackievirus A6, CVA6 | Human parainfluenza virus 4, HPIV-4 | Rhinovirus C, HRV-C |
|  |  | Coxsackievirus A10, CVA10 | Influenza A virus, IAV | Rubella virus, RuV |
|  |  | Coxsackievirus A16, CVA16 | Influenza A virus H1N1 | SARS-CoV-2 (2019-nCoV) |
|  |  | Enteric cytopathic human orphan (ECHO) virus type 18, ECHO18 | Influenza A virus H3N2 |  |
|  |  | Human Coronavirus 229E, HCoV-229E | Influenza A virus H5N1 |  |
|  |  | Human Coronavirus HKU1, HCoV-HKU1 | Influenza A virus H7N9 |  |
| Bacteria | G+bacteria | *Arcanobacterium haemolyticum, A. haemolyticum* | *Streptococcus anginosus, S. anginosus* | *Streptococcus pneumoniae, S. pneumoniae* |
|  |  | *Corynebacterium diphtheriae, C. diphtheriae* | *Streptococcus dysgalactiae, S. dysgalactiae* | *Streptococcus pyogenes, S. pyogenes* |
|  |  | *Staphylococcus aureus,* *S. aureus* | *Streptococcus intermedius, S. intermedius* | *Streptococcus agalactiae, GBS* |
|  | G−bacteria | *Acinetobacter baumannii, A. baumannii* | *Klebsiella pneumoniae, K. pneumoniae* | *Neisseria meningitidis, N. meningitidis* |
|  |  | *Bordetella pertussis, B. pertussis* | *Legionella pneumophila, L. pneumophila* | *Pseudomonas aeruginosa,* *P. aeruginosa* |
|  |  | *Fusobacterium necrophorum, F. necrophorum* | *Moraxella catarrhalis, M. catarrhalis* | *Serratia marcescens, S. marcescens* |
|  |  | *Haemophilus influenzae, H. influenzae* | *Neisseria gonorrhoeae, N. gonorrhoeae* | *Stenotrophomonas maltophilia, S. maltophilia* |
|  |  | *Listeria monocytogenes, L. monocytogenes* | *Corynebacterium ulcerans, C. ulcerans* | *Yersinia enterocolitica, Y. enterocolitica* |
|  |  | *Haemophilus influenzaeB, H.influenzaeB* |  |  |
| Mycoplasma/Chlamydia | | *Mycoplasma pneumoniae, M. pneumoniae* | *Chlamydia psittaci, C. psittaci* | *Ureaplasma urealyticum, U. urealyticum* |
|  |  | *Chlamydophila pneumoniae, C. pneumoniae* | *Chlamydia trachomatis, C. trachomatis* | *Ureaplasma parvum, U. parvum* |
|  |  | *Mycoplasma hominis, M. hominis* | *Mycoplasma genitalium, M.genitalium* |  |
| Detectable SARS-CoV-2 Genotype | | Alpha(B.1.1.7), Omicron(BA.4), Beta(B.1.351), Omicron(BA.5), Delta(B.1.617.2), Omicron(BA.5.2), Gamma(P.1), Omicron(BA.5.7), Lamda(C.37), Omicron(BA.5.11), Mu(B.1.621), Omicron(BQ.1), Omicron(B.1.1.529), Omicron(BQ.1.1), Omicron(BA.2.12.1), Omicron(BF.7), Omicron(BA.2.75), Omicron(XBB), Omicron(BN.1), Omicron(XBB.1), Omicron(BN.3), Omicron(XBB.1.5) | | |
| Macrolide Resistance Mutation in  *M. pneumoniae* | | 23S rRNA: A2063G, 23S rRNA: A2064G, 23S rRNA: A2067G, 23S rRNA: C2617G | | |
| Macrolide Resistance Mutation in  *B. pertussis* | | 23S rRNA: A2047G | | |


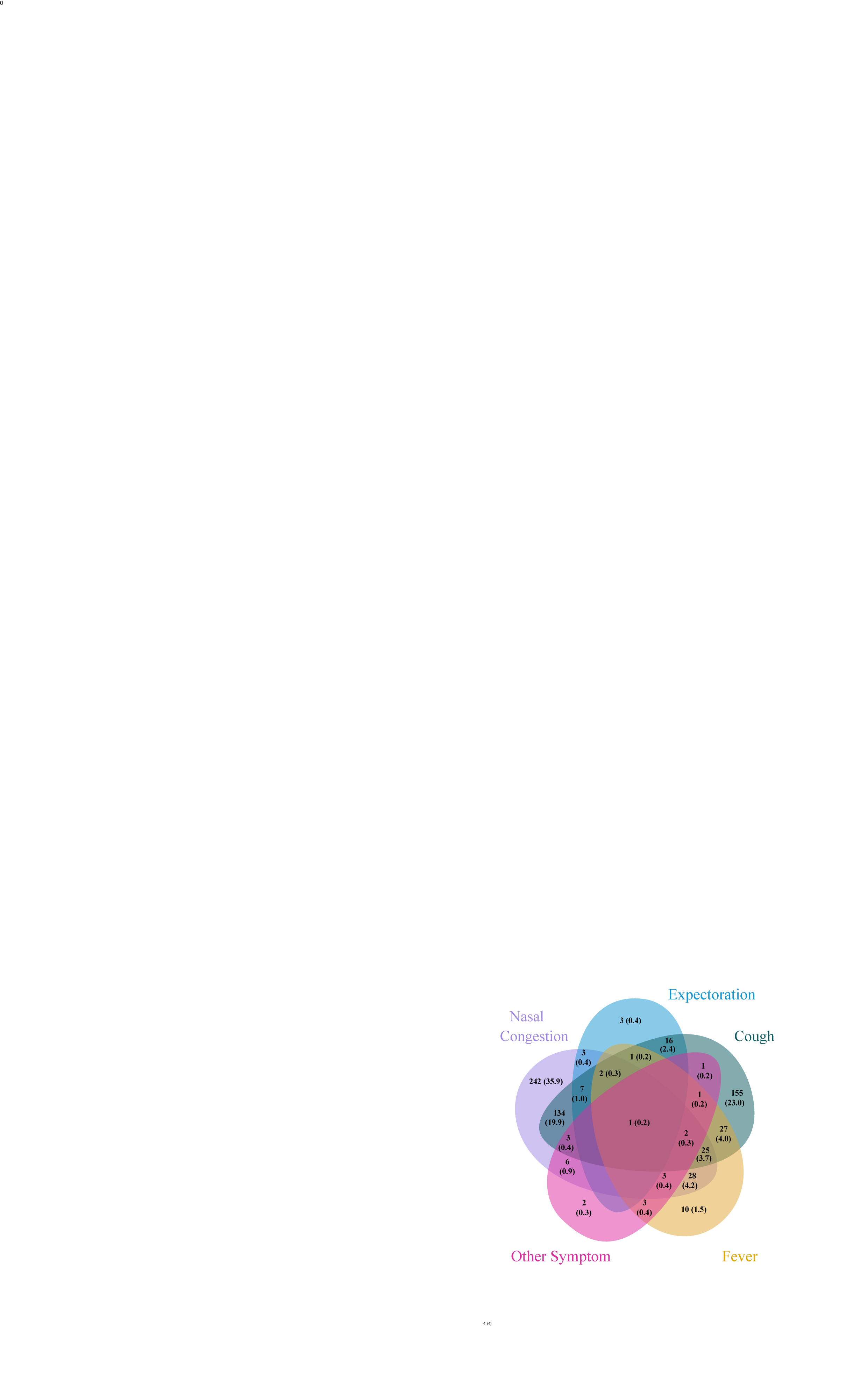
Appendix 2. Proportional venn diagram of symptom overlap seen of acute respiratory infections among infant cases (n=675).

Data are n (%), other symptoms include more than one of the following: sneezing, sore throat, wheezing, paroxysmal crying, milk vomiting, diarrhea, trachea inflammation, diarrhea, and indigestion.

Appendix 3. Positive rate of any pathogen in 675 infant case presenting with acute respiratory infections during February, 2023 to April, 2024.

|  | Positive rate, n (%) | Total | *P-value* |
| --- | --- | --- | --- |
| Overall | 636 (94.2) | 675 |  |
| Sex |  |  | 1.0 |
| Female | 286 (94.4) | 303 |  |
| Male | 350 (94.1) | 372 |  |
| Age group, month |  |  | 0.036 |
| 2-3 | 50 (86.2) | 58 |  |
| 4-6 | 95 (93.1) | 102 |  |
| 7-9 | 150 (96.2) | 156 |  |
| 10-16 | 341 (95.0) | 359 |  |

The positive rate was determined by considering the number of cases with at least one pathogen detected as the numerator, and the total number of cases that were tested as the denominator. Chi square test or Fisher’s exact test were used for comparisons for sex groups or age groups.

Appendix 4. Positive rate of any pathogen in 50 hospitalized infant cases.

|  | Positive rate, n (%) | Total | *P-value* |
| --- | --- | --- | --- |
| Overall | 31 (62.0) | 50 |  |
| Sex |  |  | 0.35 |
| Female | 17 (70.8) | 24 |  |
| Male | 14 (53.8) | 26 |  |
| Age group, month |  |  | 0.37 |
| 2-3 | 11 (52.4) | 21 |  |
| 4-6 | 20 (69.0) | 29 |  |

The positive rate was determined by considering the number of cases with at least one pathogen detected as the numerator, and the total number of cases that were tested as the denominator. Chi square test or Fisher’s exact test were used for comparisons for sex groups or age groups.

Appendix 5. Positive rate of respiratory pathogens among 50 Hospitalized infant cases by gender, age group.

| Pathogen | All cases | Sex | | *P-value* | Age group, month | | *P-value* |
| --- | --- | --- | --- | --- | --- | --- | --- |
|  |  | Male (n=26) | Female (n=24) |  | 2-3 (n=21) | 4-6 (n=29) |  |
| DNA virus |  |  |  |  |  |  |  |
| HAdV | 18 (36.0) | 6 (23.1) | 12 (50.0) | 0.092 | 5 (23.8) | 13 (44.8) | 0.22 |
| CMV | 1 (2.0) | 1 (3.9) | 0 | 1.0 | 0 (0.0) | 1 (3.5) | 1.0 |
| RNA virus |  |  |  |  |  |  |  |
| RSV | 18 (36.0) | 6 (23.1) | 12 (50.0) | 0.092 | 5 (23.8) | 13 (44.8) | 0.22 |
| InfluenzaA | 8 (16.0) | 1 (3.9) | 7 (29.2) | 0.04 | 5 (23.8) | 3 (10.3) | 0.37 |
| HPIV | 8 (16.0) | 1 (3.9) | 7 (29.2) | 0.04 | 2 (9.5) | 6 (20.7) | 0.50 |
| CVB | 8 (16.0) | 4 (15.4) | 4 (16.7) | 1.0 | 3 (14.3) | 5 (17.2) | 1.0 |
| ECHO | 8 (16.0) | 4 (15.4) | 4 (16.7) | 1.0 | 3 (14.3) | 5 (17.2) | 1.0 |
| InfluenzaB | 7 (14.0) | 1 (3.9) | 6 (25.0) | 0.081 | 4 (19.1) | 3 (10.3) | 0.64 |
| SARS-CoV-2 | 1 (2.0) | 1 (3.9) | 0 (0.0) | 1.0 | 1 (4.8) | 0 (0.0) | 0.87 |
| Rotavirus | 1 (2.0) | 1 (3.9) | 0 (0.0) | 1.0 | 0 (0.0) | 1 (3.5) | 1.0 |
| RuV | 1 (2.0) | 1 (3.9) | 0 (0.0) | 1.0 | 0 (0.0) | 1 (3.5) | 1.0 |
| G+bacteria |  |  |  |  |  |  |  |
| *V.Streptococci* | 3 (6.0) | 2 (7.7) | 1 (4.2) | 1.0 | 0 (0.0) | 3 (10.3) | 0.36 |
| *S.aureus* | 1 (2.0) | 1 (3.9) | 0 (0.0) | 1.0 | 1 (4.8) | 0 (0.0) | 0.87 |
| G−bacteria |  |  |  |  |  |  |  |
| *K.oxytoca* | 1 (2.0) | 0 (0.0) | 1 (4.2) | 0.97 | 1 (4.8) | 0 (0.0) | 0.87 |
| *E.aerogenes* | 1 (2.0) | 0 (0.0) | 1 (4.2) | 0.97 | 1 (4.8) | 0 (0.0) | 0.87 |
| *H.influenzae* | 1 (2.0) | 0 (0.0) | 1 (4.2) | 0.97 | 0 (0.0) | 1 (3.5) | 1.00 |
| *E.coli* | 1 (2.0) | 0 (0.0) | 1 (4.2) | 0.97 | 0 (0.0) | 1 (3.5) | 1.00 |
| Other pathogens |  |  |  |  |  |  |  |
| *M.pneumoniae* | 12 (24.0) | 6 (23.1) | 6 (25.0) | 1.0 | 5 (23.8) | 7 (24.1) | 1.00 |
| *C.pneumoniae* | 12 (24.0) | 6 (23.1) | 6 (25.0) | 1.0 | 3 (14.3) | 9 (31.0) | 0.30 |

Data are n (%). Chi-square test or Fisher’s exact test were used for comparisons among different groups. Positive rate was calculated by taking the positive number of each pathogen as the numerator and the total number of cases that underwent the tests for each specific pathogen as denominator.

Appendix 6. Summary of serotyping results of *S. pneumoniae* [n (%)].

| Serotype | 15A/15F | 10F/10C/33C | 10A | 11A/11D | 39 | 7C/7B/40 | 5 | 8 | 15B/C | 19F | 21 | 23A | 23B | 31 |
| --- | --- | --- | --- | --- | --- | --- | --- | --- | --- | --- | --- | --- | --- | --- |
| Strain number [n (%)] | 25 (38.5) | 15 (23.1) | 9 (13.8) | 3 (4.6) | 3 (4.6) | 2 (3.1) | 1 (1.5) | 1 (1.5) | 1 (1.5) | 1 (1.5) | 1 (1.5) | 1 (1.5) | 1 (1.5) | 1 (1.5) |

Appendix 7. Comparison of co-detection rates among infant ARI cases of different age groups by different types.

|  | co-detection, n (%) | Viral-viral, n (%) | Viral-bacterial, n (%) | Viral-other pathogens, n (%) | Bacterial-bacterial, n (%) | Bacterial-other pathogens, n (%) | Viral-bacteria-other pathogens, n (%) |
| --- | --- | --- | --- | --- | --- | --- | --- |
| Overall | 497 (73.6) | 34 (6.8) | 360 (72.4) | 5 (1.01) | 71 (14.3) | 5 (1.0) | 22 (4.4) |
| Age group, month |  |  |  |  |  |  |  |
| 2-3 | 38 (65.5) | 1 (2.6) | 32 (84.2) | 0 | 3 (7.9) | 0 | 2 (5.3) |
| 4-6 | 70 (68.6) | 5 (7.1) | 53 (75.7) | 0 | 9 (12.9) | 0 | 3 (4.3) |
| 7-9 | 136 (87.2) | 13 (9.6) | 88 (64.7) | 4 (2.94) | 18 (13.2) | 3 (2.2) | 10 (7.4) |
| 10-16 | 253 (70.3) | 15 (5.9) | 187 (73.9) | 1 (0.40) | 41 (16.2) | 2 (0.8) | 7 (2.8) |

Data are n (%) unless otherwise indicated. Percentages may not total 100 because of rounding.

Appendix 8. Coexistence patterns of respiratory pathogens in 675 infant cases of acute respiratory infections.


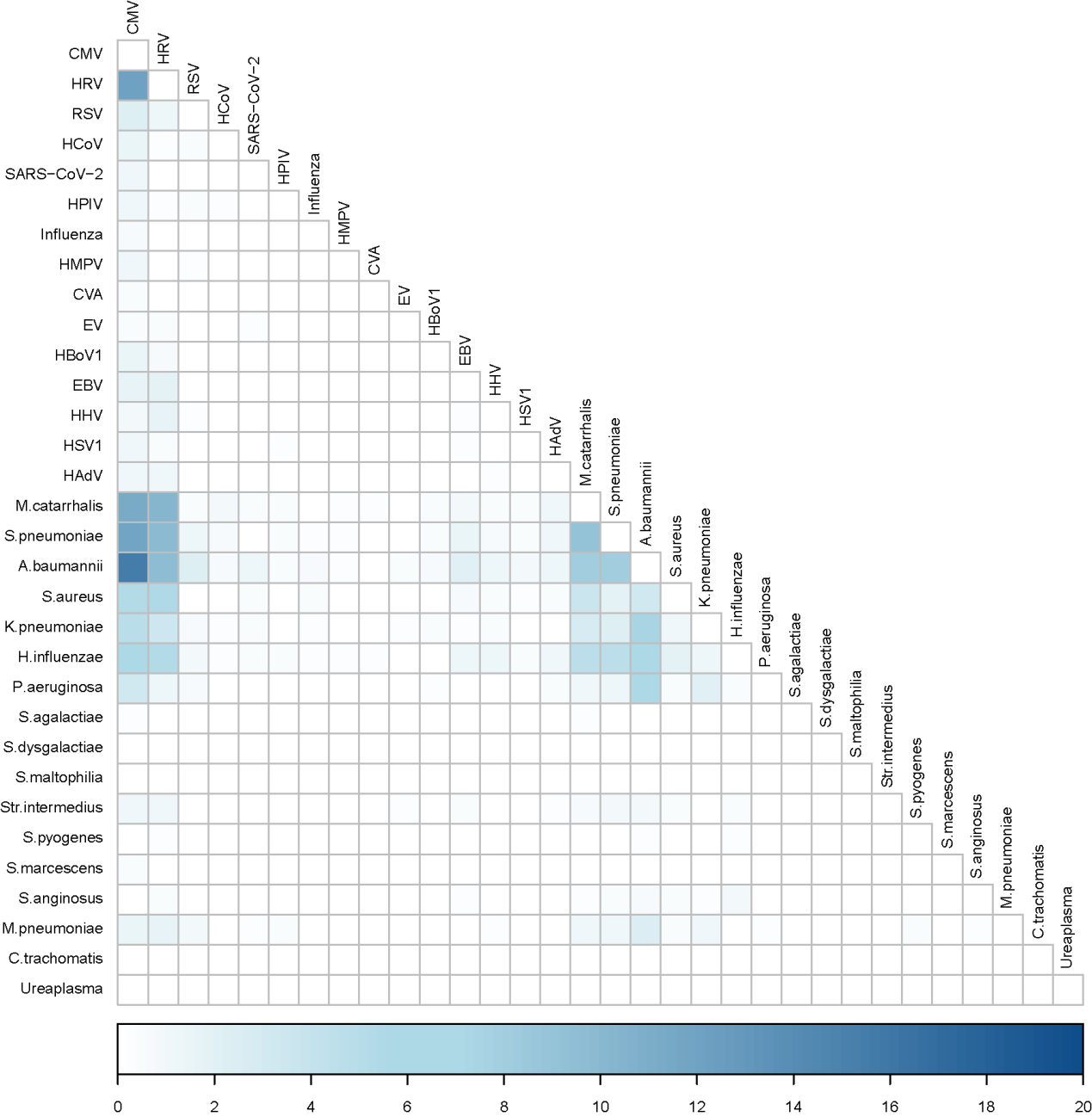


For pathogen ‘X’ and ‘Y’, numerator was the number of patients co-detected both ‘X’ and ‘Y’ and the denominator where the total number of patients who were both tested ‘X’ and ‘Y’. Darker color of the box indicates higher coexistence rates between two pathogens.

Appendix 9. Coexistence pattern of respiratory pathogens among ARI cases.


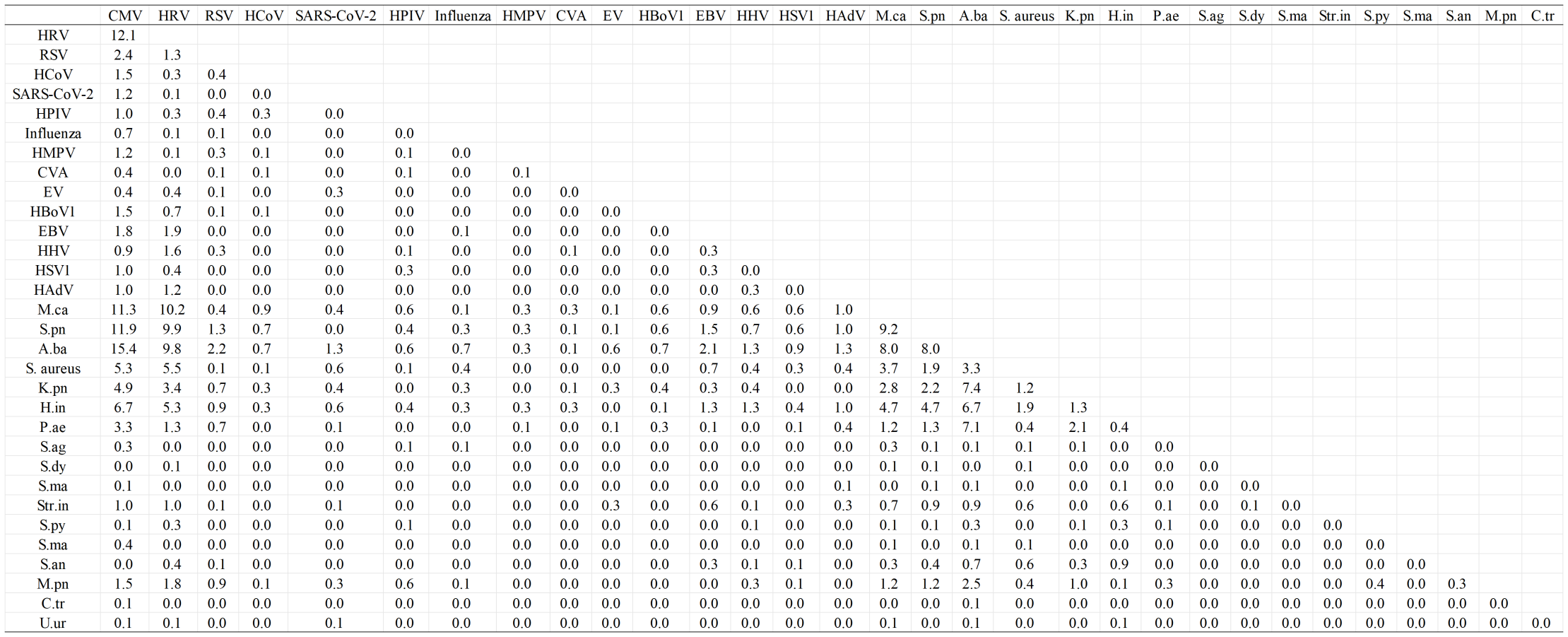
Figure shows the coexistence ratio of the pathogens. *M.ca: M. catarrhalis; S.pn: S. pneumoniae; A.ba: A. baumannii; K.pn: K. pneumoniae; H.in: H. influenzae; P.ae: Partbroninosa; S.ag: S. agalactiae; S.dy: S. dysgalactiae; S.mal: S. maltophilia; Str.in: Str. intermedius; S.py: S. pyogenes; S.mar: S. marcescens; S.an: S. anginosus; M.pn: M. pneumoniae; C.tr: C. trachomatis; U.ur: U. urealyticum*

Appendix 10. Factors related to the positive rate of CMV in infants among infant ARI cases


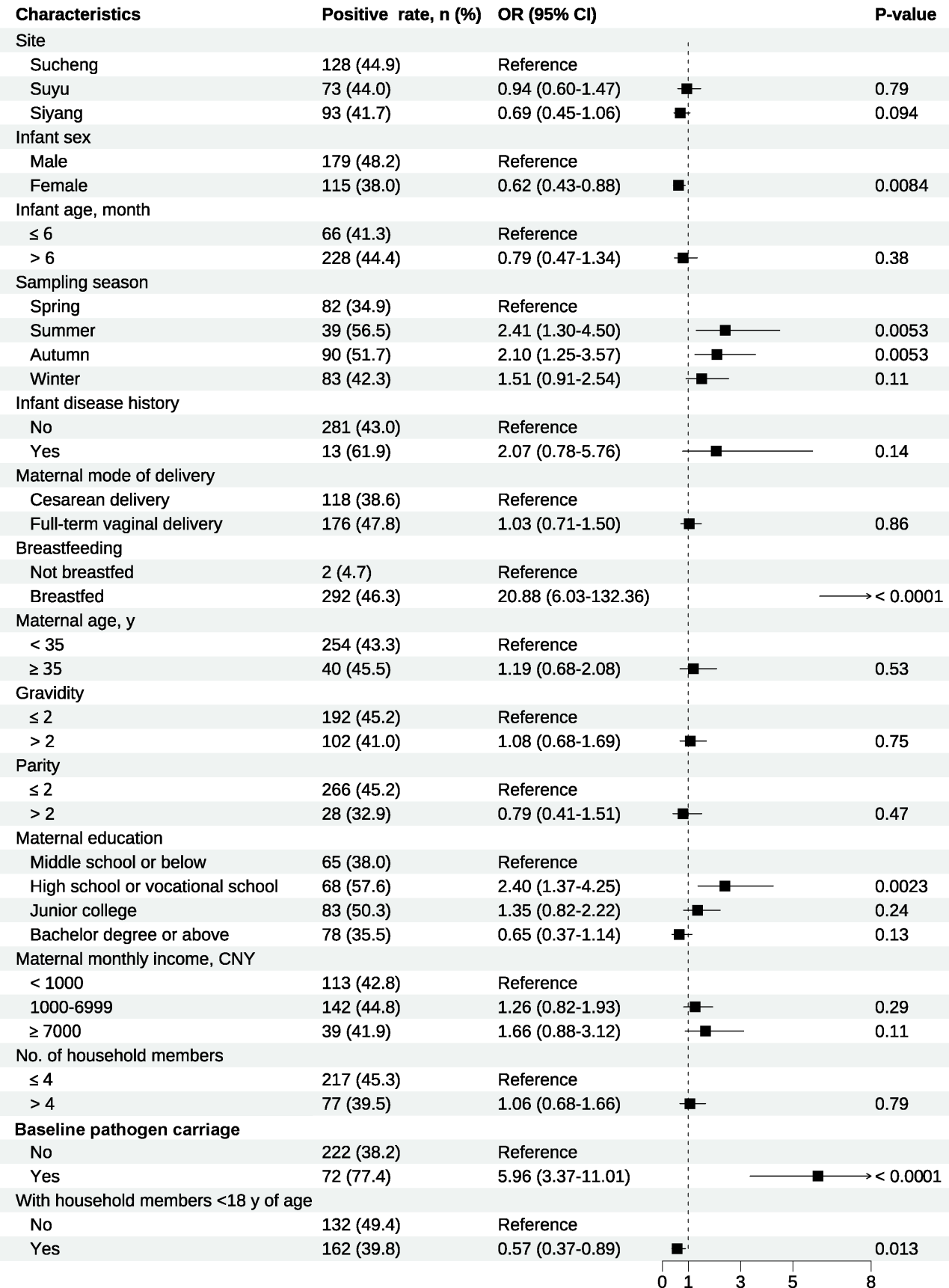


Appendix 11. Factors related to the positive rate of HRV in infants among infant ARI cases


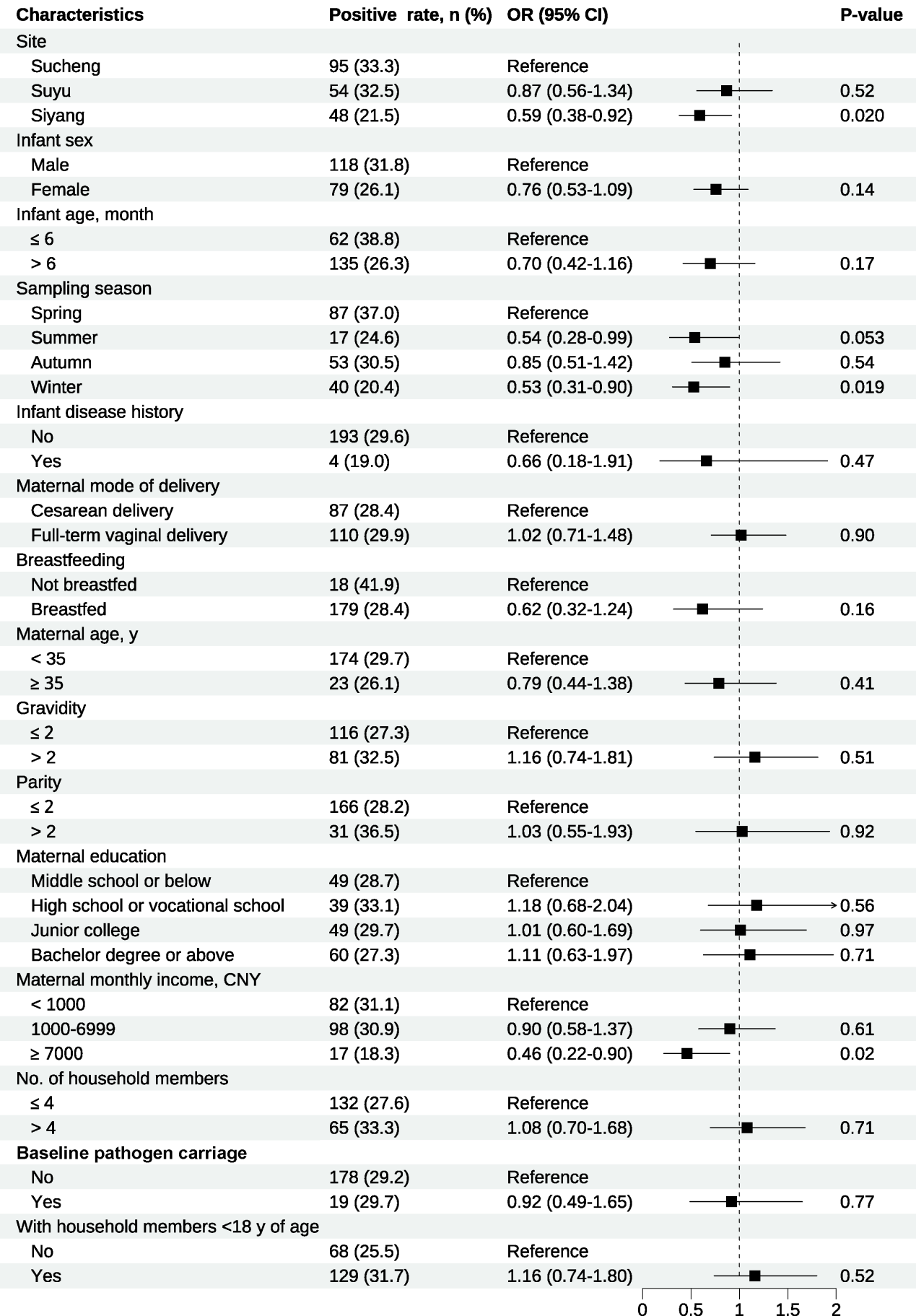


Appendix 12. Factors related to the positive rate of *S. pneumoniae* in infants among infant ARI cases


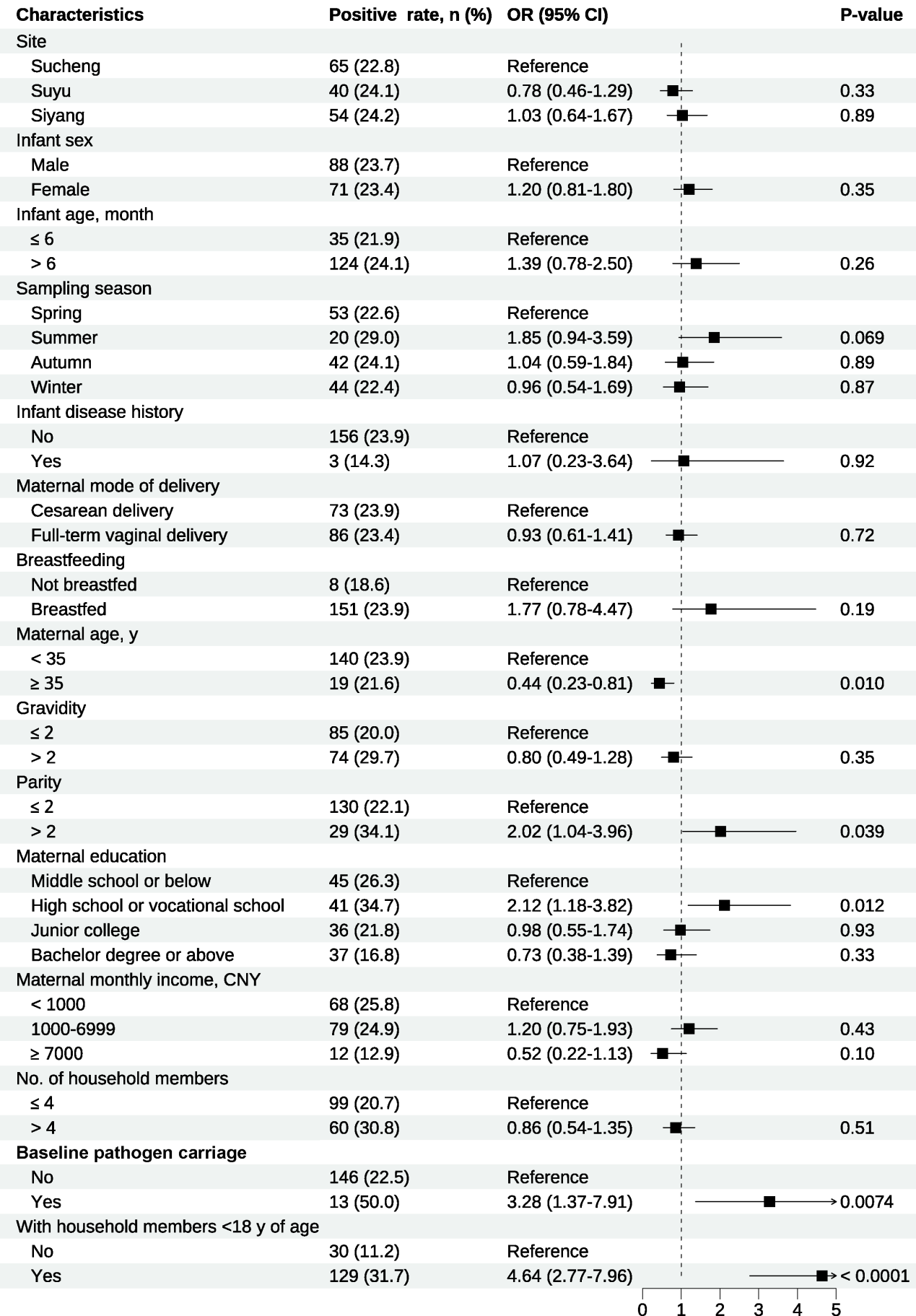


Appendix 13. Factors related to the positive rate of *S. aureus* in infants among infant ARI cases


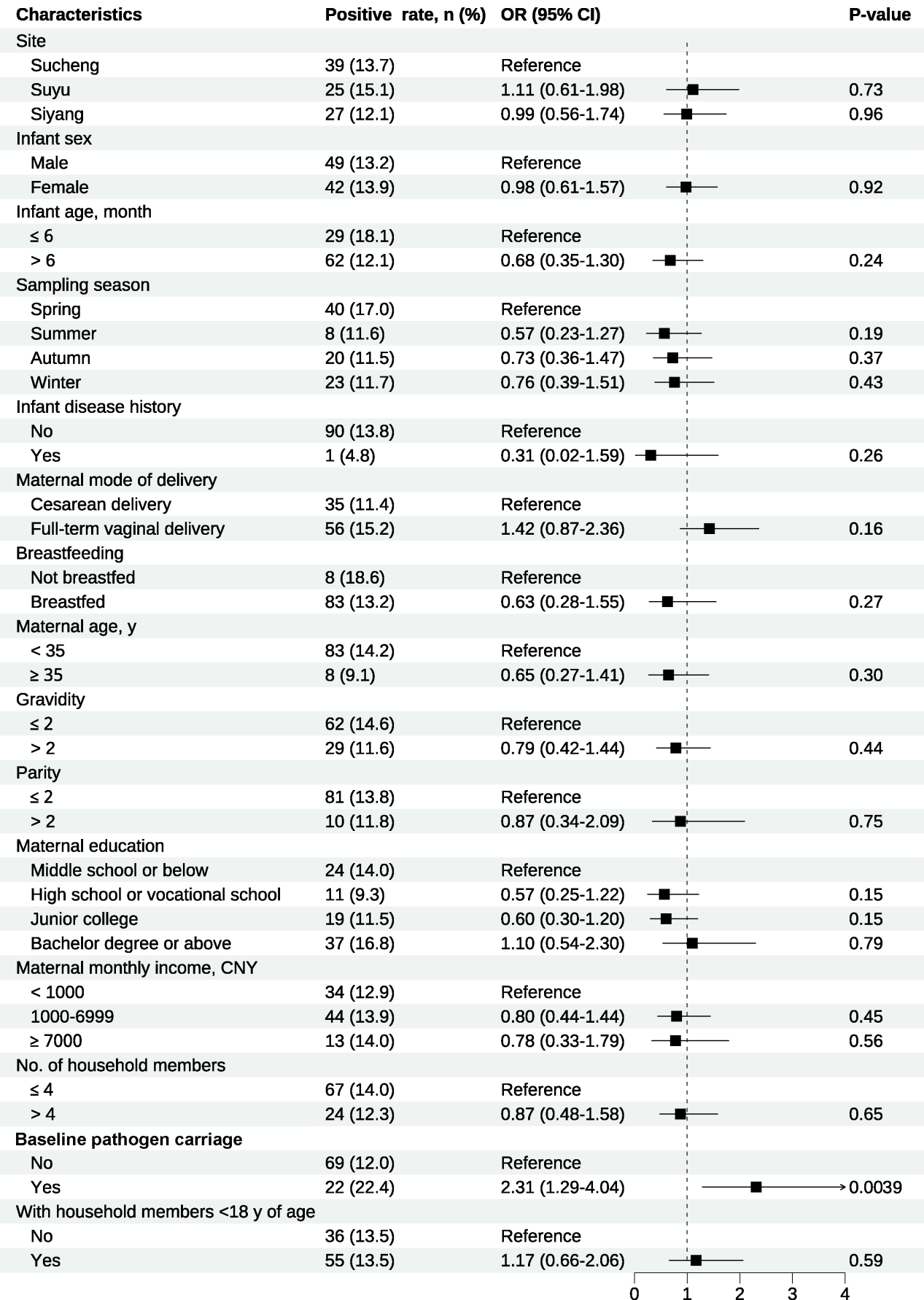


Appendix 14. Factors related to the positive rate of *A. baumannii* in infants among infant ARI cases


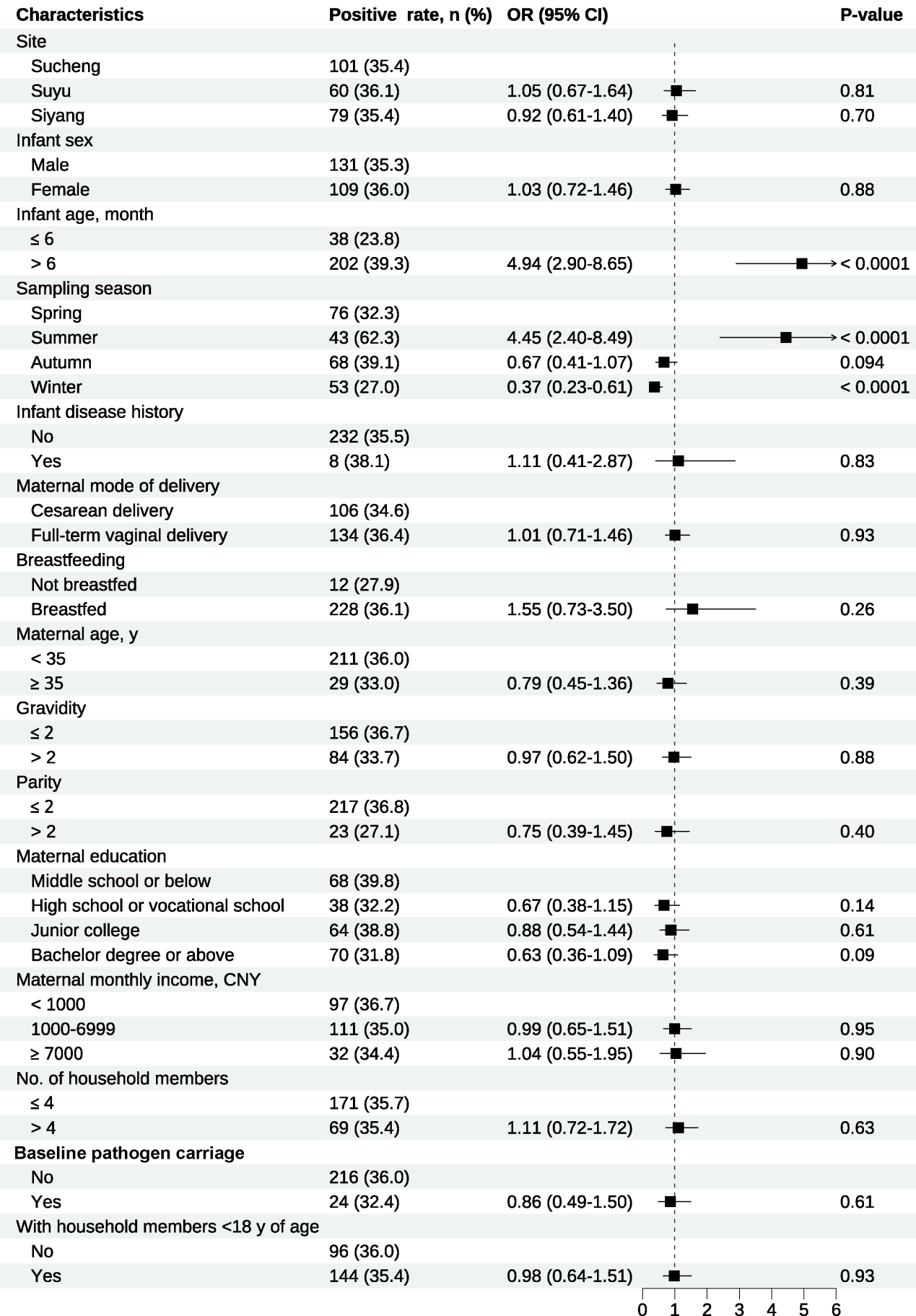


Appendix 15. Factors related to the positive rate of *M. catarrhalis* in infants among infant ARI cases


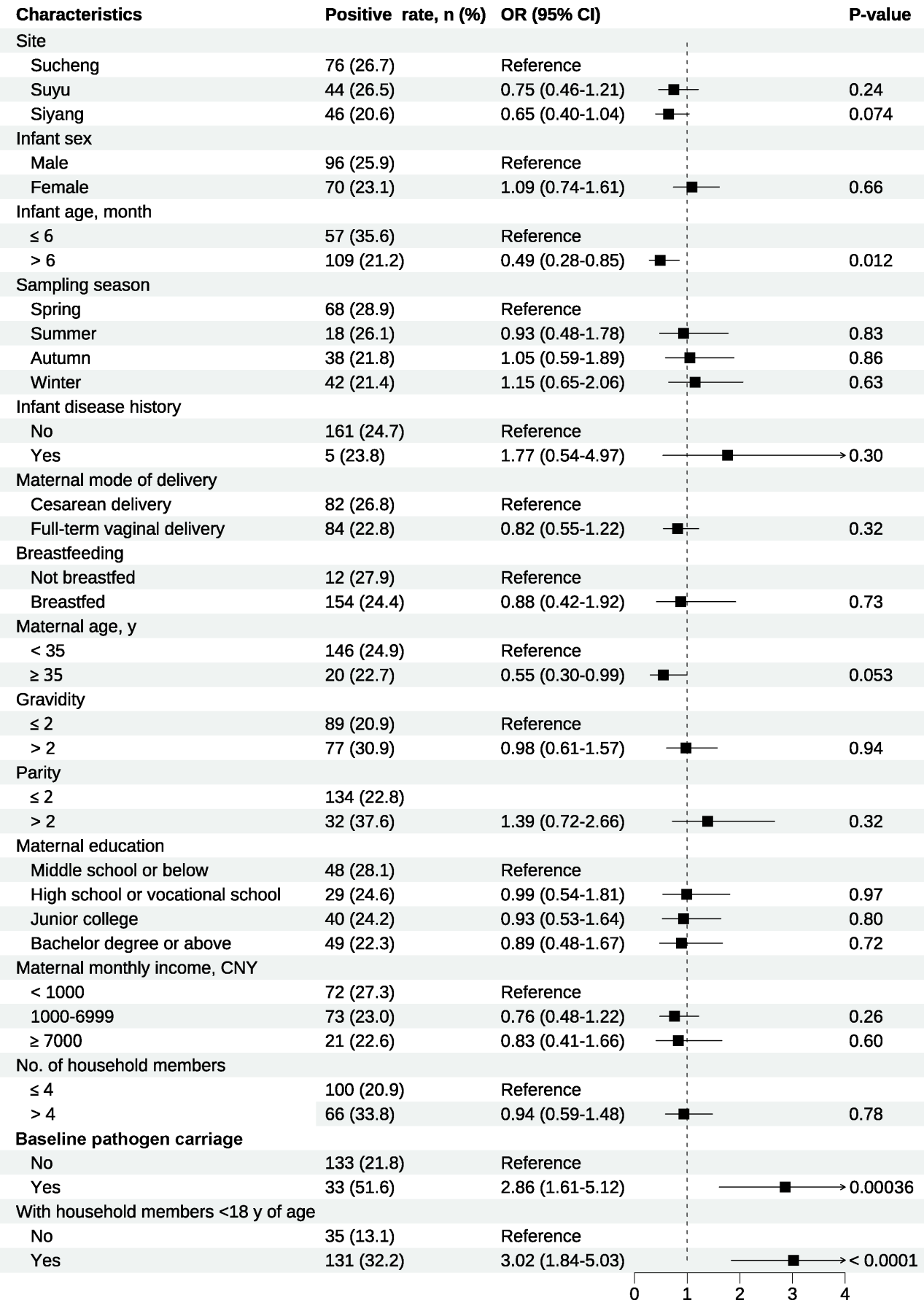


Appendix 16. Factors related to the positive rate of *M. pneumoniae* in infants among infant ARI cases


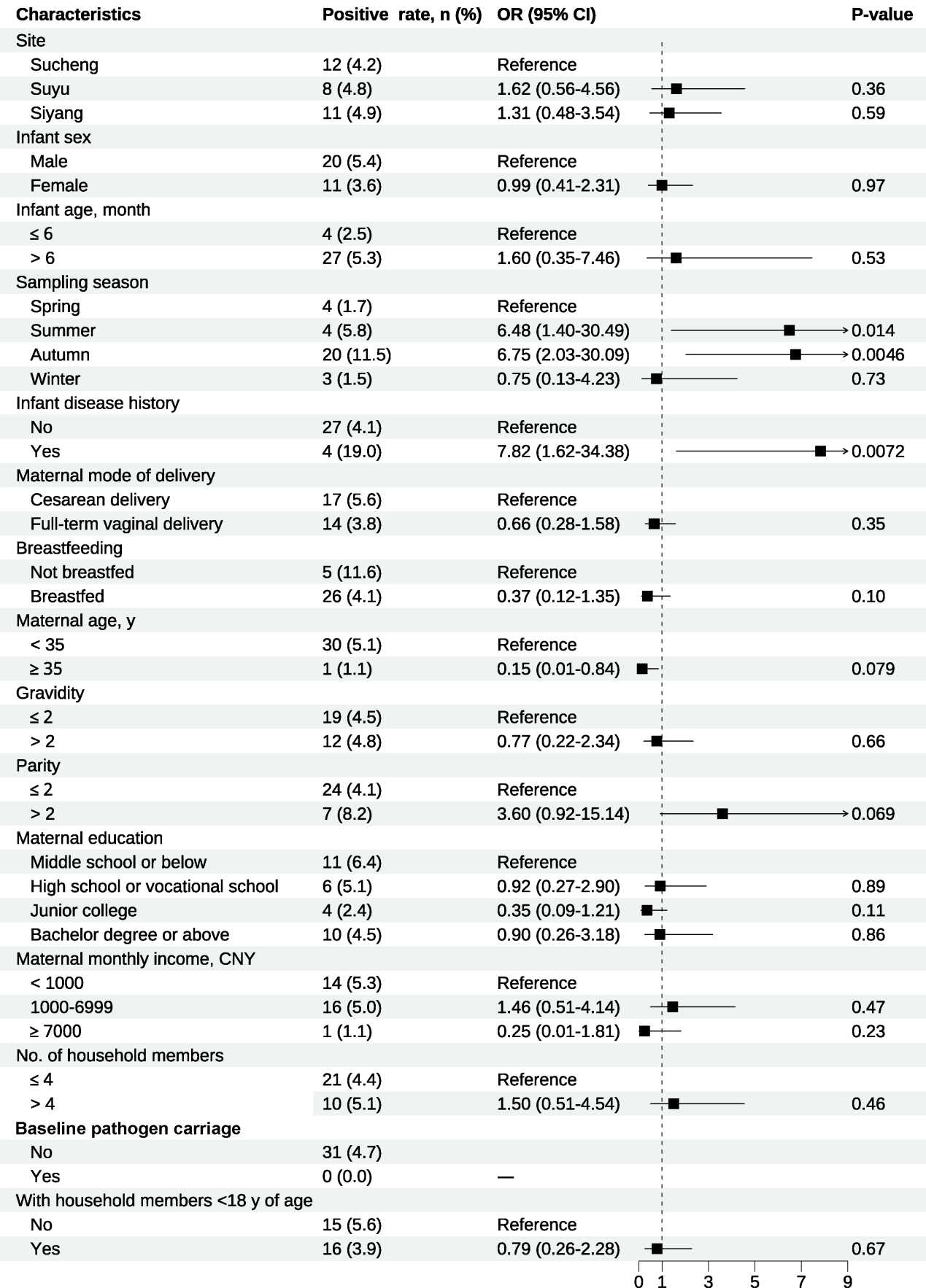


Appendix 17. Evolution of vaccine coverage Among participants in the cohort over monitoring period.


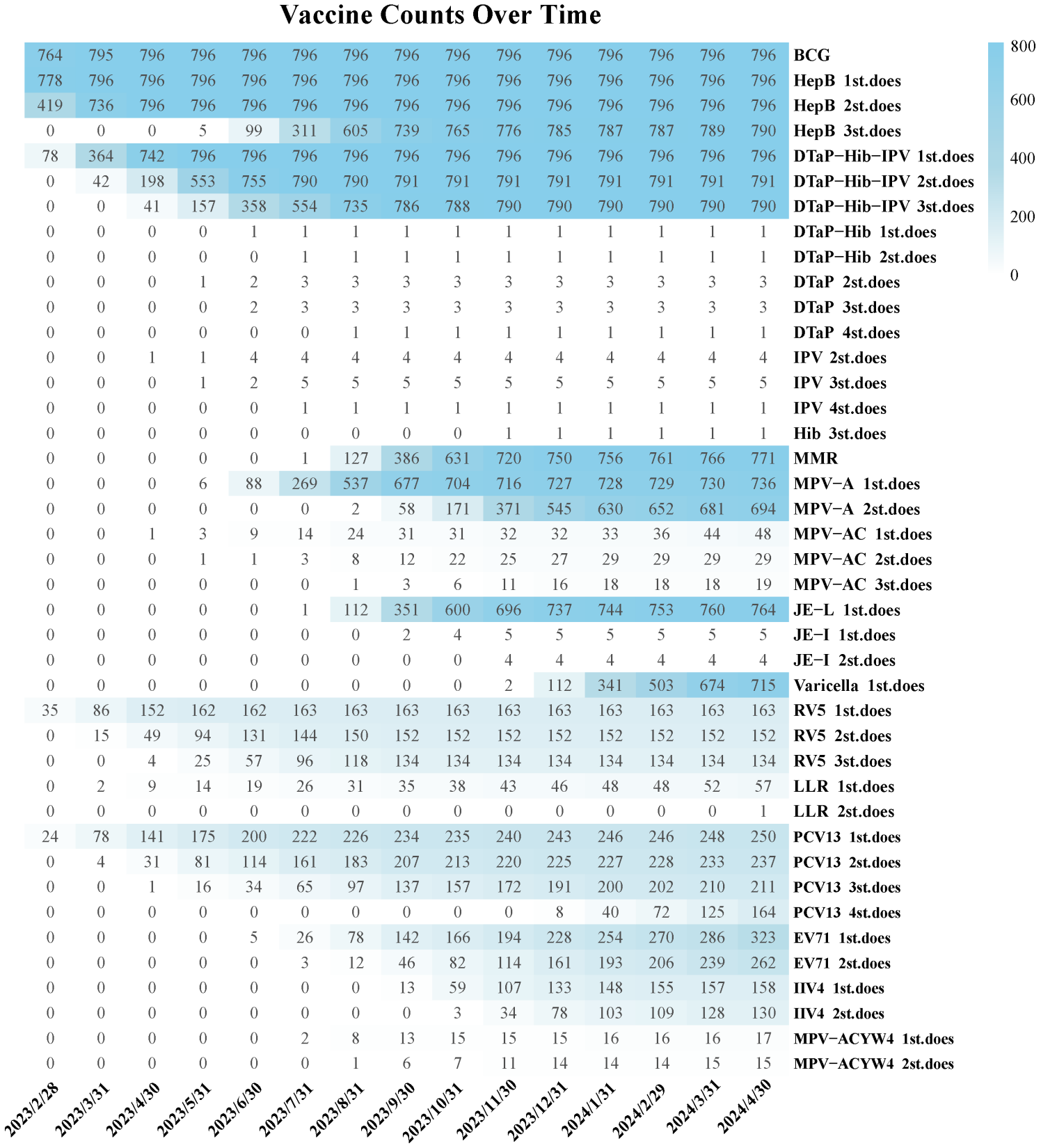


The horizontal axis represents the change in monitoring time (year/month/day), while the vertical axis indicates the types of vaccines and the number of doses administered.
